# Comparative transcriptomic analysis reveals the roles of overlapping heat-/drought-responsive genes in poplars exposed to high temperature and drought

**DOI:** 10.1038/srep43215

**Published:** 2017-02-24

**Authors:** Jingbo Jia, Jing Zhou, Wenguang Shi, Xu Cao, Jie Luo, Andrea Polle, Zhi-Bin Luo

**Affiliations:** 1 State Key Laboratory of Tree Genetics and Breeding, Key Laboratory of Silviculture of the State Forestry Administration, Research Institute of Forestry, Chinese Academy of Forestry, Beijing 100091, China; 2 College of Life Sciences, Northwest A&F University, Yangling, Shaanxi, 712100, P. R. China; 3 Büsgen-Institute, Department of Forest Botany and Tree Physiology, Georg-August University, Büsgenweg 2, 37077 Göttingen, Germany

## Abstract

High temperature (HT) and drought are both critical factors that constrain tree growth and survival under global climate change, but it is surprising that the transcriptomic reprogramming and physiological relays involved in the response to HT and/or drought remain unknown in woody plants. Thus, *Populus simonii* saplings were exposed to either ambient temperature or HT combined with sufficient watering or drought. RNA-sequencing analysis showed that a large number of genes were differentially expressed in poplar roots and leaves in response to HT and/or desiccation, but only a small number of these genes were identified as overlapping heat-/drought-responsive genes that are mainly involved in RNA regulation, transport, hormone metabolism, and stress. Furthermore, the overlapping heat-/drought-responsive genes were co-expressed and formed hierarchical genetic regulatory networks under each condition compared. HT-/drought-induced transcriptomic reprogramming is linked to physiological relays in poplar roots and leaves. For instance, HT- and/or drought-induced abscisic acid accumulation and decreases in auxin and other phytohormones corresponded well with the differential expression of a few genes involved in hormone metabolism. These results suggest that overlapping heat-/drought-responsive genes will play key roles in the transcriptional and physiological reconfiguration of poplars to HT and/or drought under future climatic scenarios.

The global temperature is predicted to rise 3–6 °C by 2100^1^. High temperature (HT) can cause heat stress in trees, thereby leading to reduced growth and development^2^. In addition to rises in the global temperature, the frequency, intensity, and duration of droughts are also anticipated to increase in the future, which may decrease forest productivity and increase tree mortality^3^,^4^. Thus, HT and drought are two major environmental constraints that hinder tree growth. Furthermore, there is increasing evidence that widespread tree mortality has been associated with rising temperatures and drought events during the last few decades^3^,^5^. Therefore, it is important to obtain a better understanding of the molecular and physiological mechanisms that underlie the responses of woody plants to HT and/or drought.

Several studies have addressed the impact of HT on molecular and physiological processes in herbaceous plants^6^, but less information is available about the responses of woody plants to this environmental cue^2^,^7^. In herbaceous plants, HT can lead to DNA damage, transcriptomic reprogramming, proteomic changes, inhibition of CO_2_ assimilation, altered phytohormone concentrations, and shifted homeostasis between reactive oxygen species (ROS) and antioxidants^6^. However, only a few studies have reported the molecular and physiological acclimation of woody plants to HT^7^,^8^,^9^. In birch (*Betula papyrifera*) and aspen (*Populus tremuloides*) trees, natural HT (36–39 °C) caused decreases in photosynthetic electron transport and triose phosphate use^10^. In the leaves of *P. euphratica* seedlings, transient HT (at 45 °C for 3 h) led to transcriptional changes that were characterized by the enrichment of Gene Ontology (GO) terms related to hormone-mediated signal, biological process regulation, and metabolic process regulation, as well as by significantly up-regulated mRNA levels for several genes encoding heat shock proteins (HSPs)^11^. It is expected that woody plants must endure HT more frequently than herbaceous plants due to their longer lifespan, and different tree species may possess distinct mechanisms for acclimation to HT. Due to the limited amount of information available about tree heat acclimation, more studies are needed to elucidate the molecular and physiological mechanisms involved.

Drought is one of the most important abiotic stressors for woody plants. Numerous studies have shown that desiccation can result in the differential expression of genes and proteins, decreased water status and photosynthate levels, activated hormonal signalling, as well as induced ROS production and antioxidant activities in trees^12^,^13^,^14^. Under natural conditions, drought often accompanies HT. The molecular and physiological mechanisms involved when exposed to drought combined with HT have been explored in herbaceous plants^15^. Thus, it has been shown that the gene expression levels and metabolic activity in *Arabidopsis* plants were modified in a distinct manner when exposed to a combination of drought and HT compared with that under each individual stress^16^,^17^. Surprisingly, few studies have addressed the effects of drought combined with HT on the molecular and physiological responses of woody plants^2^. Indeed, only one report has described the physiological and proteomic responses of poplar (*P. yunnanensis*) saplings to sustained HT (48 h) and drought^18^, where it was demonstrated that combined HT and drought had more harmful effects on poplar plantlets than each individual stress^18^. Recently, it was shown that drought-induced physiological responses and transcriptional changes in genes involved with heat stress responses, abscisic acid (ABA) biosynthesis, and sugar transport were increased by HT in the roots and leaves of *P. alba* × *P. tremula* var. *glandulosa* saplings^7^. These results demonstrate that it is necessary to investigate the molecular and physiological mechanisms that are activated in woody plants in response to combined drought and HT in the context of future climatic conditions.

*Populus* species are model trees for studying the molecular and physiological mechanisms that are activated in woody plants in response to HT and/or drought^7^,^18^. *P. simonii* often grows in semi-arid and nutrient-poor regions^19^, which may be affected by HT combined with drought in future climatic conditions. To examine the genome-wide transcriptional and physiological regulation of poplars to HT and/or drought, we exposed *P. simonii* saplings to either ambient temperature (AT) or HT combined with sufficient irrigation or drought. In the present study, we aimed to (i) characterize genome-wide transcriptional reprogramming occurring in poplar roots and leaves in response to HT and/or drought; and (ii) test the hypothesis that HT- and/or drought-triggered physiological changes can be linked with the transcriptomic configuration in the roots and leaves of poplars. To achieve both goals, we performed RNA-sequencing analyses and identified overlapping heat-/drought-responsive genes in the roots and leaves of *P. simonii*. We also characterized various physiological changes (water status, photosynthesis, stable carbon/oxygen isotope compositions, non-structural carbohydrates, phytohormones, ROS, and antioxidants) and their links to transcriptomic reprogramming in poplars in response to HT and/or drought. Our results provide insights that may facilitate the breeding of woody plants with tolerance to HT in combined with drought under future climatic scenarios.

## Results and Discussion

### Overlapping heat/drought responses in poplar root and leaf transcriptomes

**Figure 1:**
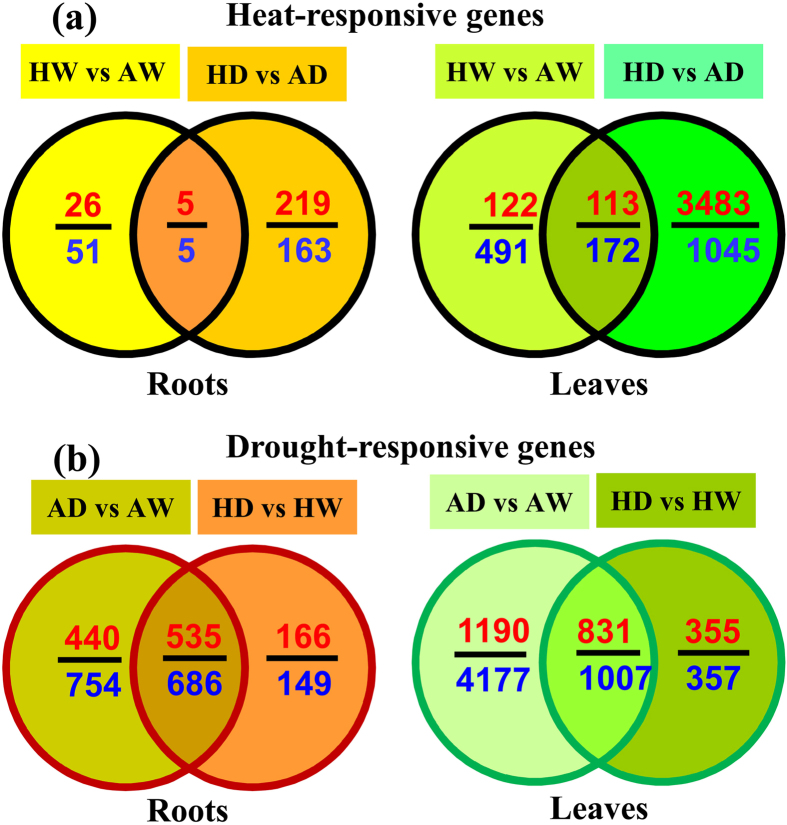
Significantly differentially expressed genes in the roots and leaves of *P. simonii* treated with either ambient (A) or high (H) temperatures combined with one of two watering regimes (well-watered (W) or drought (D)). The upper and lower numbers in each fraction indicate the numbers of up- and down-regulated genes, respectively. The overlapping genes under HT (**a**) were defined as heat responsive genes, and the overlapping genes under drought stress (**b**) were defined as drought responsive genes. Detailed information about the significantly differentially expressed genes and overlapping genes under each condition compared is presented in Supplementary Tables S2 and S3, respectively.

To characterize the transcriptomic regulation of genes in the roots and leaves of *P. simonii* in response to HT and/or drought, we performed genome-wide RNA-sequencing analyses. After filtering and sequence trimming (Table S1), the clear reads were mapped to the reference genome of *P. trichocarpa* and used to identify significantly differentially expressed genes in the roots and leaves in response to heat under well-watered (HW vs AW) or drought (HD vs AD) conditions (Fig. 1a, Table S2). In the roots of *P. simonii*, HT yielded 31 up- and 56 down-regulated genes under well-watered conditions, and 224 up- and 168 down-regulated genes under drought conditions (Fig. 1a, Table S2). Among these genes, five up- and five down-regulated genes were overlapped between HW vs AW and HD vs AD, which were defined as the overlapping heat-responsive genes in the roots (Fig. 1a, Table S3). In *P. simonii* leaves, HT increased the mRNA levels of 235 genes and decreased the transcript levels of 663 genes under HW vs AW, and the up-regulation of 3596 genes and down-regulation of 1217 genes under HD vs AD (Fig. 1a, Table S2). In total, 285 genes exhibited overlapping heat responses with 113 up- and 172 down-regulated genes in poplar leaves between HW vs AW and HD vs AD (Fig. 1a, Table S3). In addition to the overlapping heat responsive genes that exhibited consistent transcriptional regulation in the two conditions compared, 11 genes in the roots and 165 genes in the leaves that exhibited opposing transcriptional regulation responses between HW vs AW and HD vs AD were overlapping (Table S4). The results of RNA-sequencing analysis were validated by analyzing several randomly selected genes using RT-qPCR (Fig. S1).

In contrast to other studies where HT was applied only for a few hours^11^,^20^, the HT treatments applied in the current study lasted for a relatively long time period of 8 days, which enabled transcriptomic and physiological reconfiguration in response to HT. The transcriptional regulation of a large number of genes in the roots and leaves of *P. simonii* when exposed to HT under both watering regimes demonstrated that transcriptomic reprogramming occurred in response to long-term HT. Moreover, a greater number of differentially expressed genes were induced in the roots and leaves of *P. simonii* by HT under drought conditions compared with HT under well-watered conditions (392 vs 84 genes in the roots and 4813 vs 898 genes in the leaves), which suggests that the impact of HT on transcriptional regulation was exacerbated by desiccation. Similarly, the effects of HT on proteomic regulation are exacerbated by drought in *P. yunnanensis* leaves^18^. In herbaceous plants, the effects of HT on transcriptomic reprogramming are also aggravated by water deficit^21^,^22^. In the present study, HT induced the differential expression of a large number of genes in poplar roots and leaves under both watering conditions, but only a small fraction of these genes overlapped between HW vs AW and HD vs AD, which agrees with previous results obtained in *Arabidopsis*^16^,^17^. These findings suggest that it is also important to consider the effects of HT on transcriptional regulation in trees under both well-watered and drought conditions.

In the roots, drought led to 975 up-regulated genes and 1440 down-regulated genes under AD vs AW, and 701 up-regulated genes and 835 down-regulated genes under HD vs HW (Fig. 1b, Table S2). Under both conditions (AD vs AW and HD vs HW), 1221 drought-responsive genes overlapped (535 up- and 686 down-regulated) in poplar roots (Fig. 1b, Table S3). In the leaves, desiccation increased the mRNA levels of 2021 genes and decreased the transcript levels of 5184 genes under AD vs AW, and 1186 up-regulated and 1364 down-regulated genes under HD vs HW (Fig. 1b, Table S2). Under AD vs AW and HD vs HW, the overlapping drought-responsive genes comprised 831 up-regulated and 1007 down-regulated genes in the leaves (Fig. 1b, Table S3). In addition to the overlapping drought-responsive genes with consistent transcriptional regulation in the two conditions compared, eight genes in the roots and 80 genes in the leaves that exhibited opposite differences in their transcriptional regulation between both conditions (AD vs AW and HD vs HW) were overlapping (Table S4).

Drought is a key abiotic stress that affects tree growth and survival in nature, and thus many studies have investigated transcriptional regulation in woody plants exposed to water deficit^23^,^24^,^25^,^26^,^27^,^28^,^29^. However, little is known about transcriptomic reconfiguration in trees exposed to severe drought combined with HT. In this study, we found that desiccation induced a large number of differentially expressed genes in the roots and leaves of *P. simonii* when exposed to either AT or HT. Moreover, we identified the overlapping drought-responsive genes between AD vs AW and HD vs HW in poplar roots/leaves because these genes could play key roles in modulating physiological relays in poplars in responses to water deficit under either AT or HT.

### Overlapping heat-/drought-responsive genes are mainly involved in RNA regulation, transport, hormone metabolism, and stress

**Figure 2:**
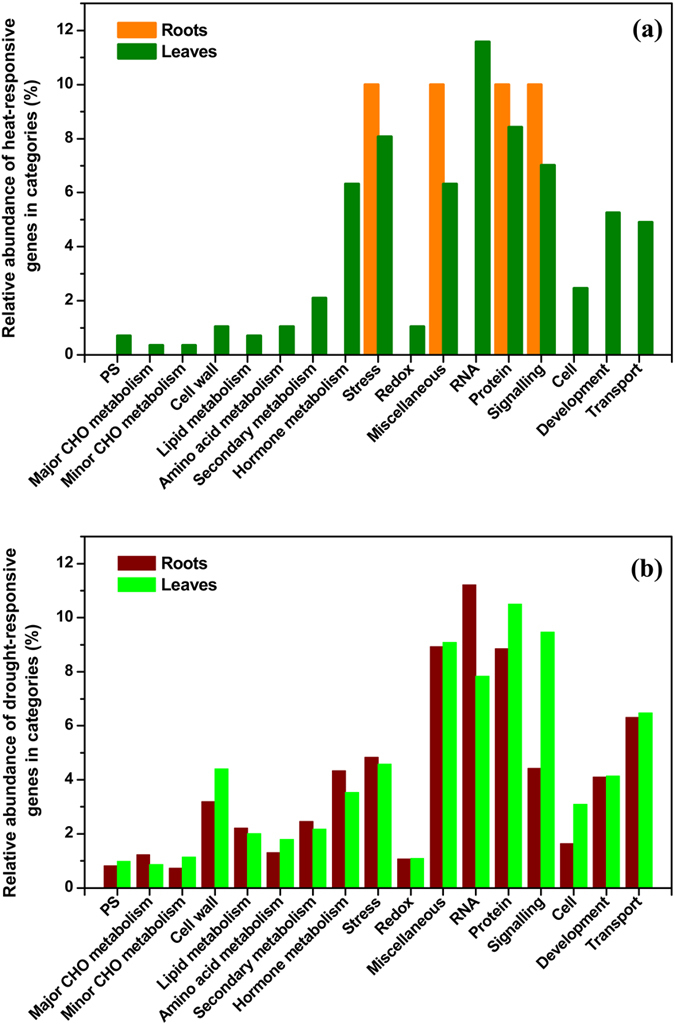
Relative abundances among the categories of overlapping heat-responsive (**a**) or drought-responsive (**b**) genes in the roots and leaves of *P. simonii* assigned by MapMan analysis. Detailed information about each category is presented in Supplementary Table S5.

To further categorize the functions of the overlapping heat-/drought-responsive genes in *P. simonii*, these poplar gene IDs were selected and analyzed using MapMan (Table S5). Only 10 overlapping heat-responsive genes were found in poplar roots and only four functional categories were related to these genes (Fig. 2a, Table S5). One gene (Potri.010G150800) that putatively encodes HS1 (heat stable protein 1) was down-regulated in *P. simonii* roots under HT, irrespective of the watering regime (Table S5). HS1 is characterized by its antifungal and antibacterial features in *A. thaliana*^30^, and it is homologous to SP1 in *P. tremula (PtSP1*), where the abundance of this protein is down-regulated under heat stress^31^. These results suggest that *HS1* probably acts as a transcription factor (TF) and regulates the heat stress responses in poplar roots.

In *P. simonii* leaves, most of the overlapping heat-responsive genes were related to the categories of RNA regulation, transport, as well as hormone metabolism and stress (Fig. 2a, Table S5). In the RNA regulation category, the expression levels of TFs including *ERF1* and *HSFA2* were down-regulated, whereas that of *ZFHD1* was over-expressed in poplar leaves under HT (Table S5). *ERF1* encodes a member of the ERF/AP2 TF family and the protein contains an AP2 domain, which is phosphorylated *in planta*^32^. In a recent study, ERF family members were characterized according to their roles in regulating plant responses to HT^33^. Heat Stress Factors (HSFs) play central roles in plant responses to heat stress. HSFA2 is a major HSF in plants^34^. Zinc finger homeodomain 1 (ZFHD1) binds to *early responsive to dehydration stress 1 (ERD1*) and its transcript level is induced by ABA in *A. thaliana*^35^. Similarly, the enhanced transcript levels of *ZFHD1* in well-watered and drought-stressed *P. simonii* leaves under HT agreed with the HT-induced ABA levels in poplar leaves.

In the transport category, the mRNA levels of several genes such as *delta tonoplast intrinsic protein 2;1 (TIP2;1*) were induced, whereas the transcript levels of a few genes including *sugar transport protein 13 (STP13*) were decreased in poplar leaves under HT (Table S5). *TIP2;1* encodes a delta tonoplast intrinsic protein, which functions as a water channel and ammonia (NH_3_) transporter in *A. thaliana*^36^. The enhanced transcript levels of *TIP2; 1* in well-watered and drought-stressed *P. simonii* leaves under HT suggest increased water transport between the vacuole and cytosol in HT-treated poplar leaves. *STP13* encodes a protein with a high-affinity hexose-specific/H^+^ symporter activity, which is involved in glucose import into the cytosol in plants^37^. The decreased mRNA levels of *STP13* in HT-treated poplar leaves under both watering regimes suggest that cytosolic glucose import is probably inhibited in HT-exposed poplar leaves.

In the hormone metabolism category, the expression levels of genes encoding an ABA-responsive protein and a member of ethylene response factor (ERF) subfamily B-3 were highly overexpressed, whereas the mRNA levels of genes including *drought hypersensitive 2, jasmonate-Zim-domain protein 1 (JAZ1*) and *lipoxygenase 3 (LOX3*) were reduced in poplar leaves under HT (Table S5). Phytohormones are essential for the signaling of plants in response to HTs and for regulating molecular and physiological processes during acclimation to heat stress^38^. Changes in the concentrations of plant hormones such as ABA, indole-3-acetic acid (IAA), salicylic acid (SA), and jasmonic acid (JA) have been observed in *P. alba* × *P. tremula* var. *glandulosa*^7^, *P. simonii* (see below), and other herbaceous plants when exposed to HT^39^. The differential expression of genes involved in plant hormone metabolism in HT-exposed poplar leaves may be responsible for changes in the foliar phytohormone concentrations.

In the stress category, the transcript level of a gene encoding an SPX1 involved in phosphate metabolism was up-regulated, whereas the mRNA levels of four genes encoding HSPs (two homologues of HSP70, HSP17.4, and HSP81.2) were down-regulated in poplar leaves under HT (Table S5). In *A. thaliana*, the transcript level of *AtSPX1* is responsive to phosphate and phosphite in both the roots and shoots^40^. Interestingly, a previous study indicated that OsSPX1 may play a role in linking cold stress and phosphate starvation signalling^41^. These results indicate that *SPX1* may be involved in heat stress signalling in poplar leaves exposed to HT. HT can induce heat stress in plants, thereby leading to protein denaturation. To acclimate to heat stress, plant cells often accumulate HSPs, which can act as molecular chaperones during protein folding in order to preserve the stability and functionality of proteins^42^. The transcriptional down-regulation of genes encoding HSP70, HSP81.2, and HSP17.4 in HT-treated *P. simonii* leaves suggests that these *HSPs* probably do not function as molecular chaperones, but instead they have other functions in poplar leaves during the response to HT. Transcriptional down-regulation of *HSPs* has also been observed in the HT-exposed leaves of *P. alba* × *P. tremula* var. *glandulosa*^7^.

Similarly, the overlapping drought-responsive genes in poplar roots and leaves were mainly enriched in the RNA regulation, transport, hormone metabolism, and stress categories (Fig. 2b, Table S5). In the RNA regulation category, the expression levels of several TFs including *RESPONSIVE TO DESICCATION 26 (RD26*) and *ABA REPRESSOR1 (ABR1*) were highly up-regulated, whereas those of TFs such as *salt tolerance zinc finger (STZ*) and *ABA RESPONSIVE ELEMENTS-BINDING FACTOR 2 (ABF2*) were severely down-regulated, in poplar roots when exposed to drought under either AT or HT (Table S5). In poplar leaves, a number of TFs such as *RD26, ABR1* and *MYB108* were highly up-regulated, but several TFs including *NAC SECONDARY WALL THICKENING PROMOTING FACTOR1 (NST1*), *HSF family* member (*HSFA6A*), and *WRKY33* were severely down-regulated in poplar leaves when exposed to drought (Table S5). TFs play essential roles in regulating the transcriptional responses of plants to drought and a number of TFs, including *MYB*s, *WRKY*s, and *HB*s, have been identified in woody plants during acclimation to water deficit^12^,^28^,^43^. *RD26* acts as a transcriptional activator in ABA-mediated dehydration response and it is involved in ABA-induced leaf senescence^44^. *ABR1* is required for priming cell death and ROS production, and disruption of *ABR1* leads to hypersensitive responses to ABA during seed germination and root growth in *Arabidopsis*^45^. In the current study, the differential expression of TFs, such as *RD26, ABR1*, and *MYB108*, in the roots and leaves of *P. simonii* in response to water deprivation suggests that these TFs play important roles in transcriptomic reconfiguration in poplars exposed to water deficit under either AT or HT.

In the transport category, the expression levels of genes encoding sugar (e.g., TMT2, STP13, and SUC2) and nitrogen (e.g., AMT1;1, NRT1.1, NRT1.2, and NRT2.5) transporters were affected in both the roots and leaves of *P. simonii* (Table S5). For instance, TMT2 is involved in glucose and fructose transport in the tonoplast^46^, while STP13 participates in monosaccharide transport and stress responses in *Arabidopsis*^37^. *SUC2* encodes a high-affinity transporter, which is essential for sucrose phloem loading, and it contributes to increased C export to the roots in drought-treated *Arabidopsis*^47^. The up-regulation of *TMT2, STP13,* and *SUC2* in the roots of *P. simonii* under either AT or HT in drought conditions corresponded well with the drought-induced concentrations of glucose, inositol, mannitol, and sucrose in poplar roots (see below), while the down-regulated genes such as *PMT2, STP7*, and *NRT1.2* suggests that the transport of nutrients and metabolites is probably reduced in drought-treated poplar leaves. The transport of nutrients and metabolites is critical for plant cell survival under drought conditions. Our results agree with a previous report that the transcriptional regulation of several genes encoding transporters for sugars, sugar alcohols, and other nutrients occurs in *P. alba* × *P. tremula* var. *glandulosa* saplings when exposed to drought under either AT or HT^7^.

In the hormone metabolism category, the mRNA expression levels of genes encoding proteins related to ABA, including ABA1, NCED3 and ABF2 were up-regulated in drought-exposed roots (Table S5). By contrast, the transcript levels of most genes involved with auxin metabolism were decreased in drought-stressed roots (Table S5). In terms of jasmonate metabolism, the mRNA levels of four genes encoding LOX1, an allene oxide synthase, allene oxide cyclase 3 (AOC3), and JAZ3 were decreased in poplar roots without watering (Table S5). In drought-treated poplar leaves, the transcript levels of three genes involved in ABA metabolism were increased, i.e., *NCED 3, ABF2*, and a *PP2C family protein* (Table S5). Moreover, the expression levels of genes involved with auxin metabolism encoding GH3.1/GH3.2/GH3.6 were highly up-regulated in dehydrated poplar leaves (Table S5). Plant hormones, such as ABA, IAA, and JA, play important roles in drought stress perception and acclimation^48^. Changes in the phytohormone concentrations and transcript levels of genes involved with hormone metabolism have been documented in woody plants exposed to drought^7^,^26^,^49^. In the drought-exposed roots and leaves of *P. simonii*, the differentially expressed genes implicated in hormone metabolism, such as *NCED3, GH3.1*, and *JAZ3*, were in good agreement with the changes in phytohormonal concentrations (see below), thereby suggesting that transcriptional regulation plays an underlying role in changes in the phytohormone levels in poplars in response to desiccation under either AT or HT. For instance, NCED3 is a key enzyme in the ABA biosynthetic pathway in plants^50^. The increased *NCED3* transcript levels corresponded well with the marked elevated ABA concentrations in the roots and leaves of *P. simonii* when treated with drought under either AT or HT, which is consistent with previous findings in *P. alba* × *P. tremula* var. *glandulosa*^7^.

In the stress category, the mRNA levels of genes including *HSP20* and *response to desiccation 2 (RD2*) were highly up-regulated in poplar roots treated with desiccation (Table S5). In dehydrated poplar leaves, the mRNA levels of genes encoding HEAT SHOCK TRANSCRIPTION FACTOR A4A (HSFA4A), a chitinase, and a pathogenesis-related thaumatin superfamily protein were induced, whereas the transcript levels of three genes related to HSPs were decreased (Table S5). Plants must activate their stress responses during acclimation to drought and the transcriptional regulation of numerous genes involved in stress responses has been reported in drought-exposed woody plants^12^. *HSP20* is responsive to heat stress in plants^51^. The transcript levels of the *RD2* gene increase in plant roots and leaves in response to water deprivation^52^. *HSFA4A* encodes a member of the HSF family and it provides a substrate for the MPK3/MPK6 signalling to regulate stress responses in *Arabidopsis*^53^. Transcriptional up-regulation of *HSP*s has also been observed in the roots and leaves of *P. alba* × *P. tremula* var. *glandulosa* when treated with water deprivation under either AT or HT^7^. Similarly, the induced transcript levels of *HSP20, RD2* and *HSFA4A* in the roots and/or leaves of *P. simonii* when exposed to water deficit suggest that the transcriptional regulation of these stress-responsive genes is important for poplars during the response to drought under either AT or HT.

In addition to the heat-/drought-responsive genes that exhibited consistent transcriptional regulation in the conditions compared (Table S3), the heat-/drought-responsive overlapping genes with opposing transcriptional regulatory responses in the conditions compared (Table S4) were also visualized using MapMan. HT led to decreased transcript levels of several aquaporin genes including three *plasma membrane intrinsic protein*s (*PIP*s) and six *tonoplast intrinsic protein*s (*TIP*s) under well-watered conditions whereas the mRNA levels of these genes increased under drought in poplar roots. Similarly, HT reduced the mRNA levels of *PIP2;2* and *TIP4;1* under well-watered conditions whereas the transcript levels of these genes increased under drought in the leaves of *P. simonii*. Similar to these results, the expression patterns of ABA-responsive gene markers also tended to be inhibited by HT under well-watered conditions, with the down-regulation of *ABF2* and *PP2C*, and the up-regulation of genes encoding PYR1-like proteins in roots, as well as the down-regulation of *NCED3, HB-7*, and *PP2C*s in the leaves (Table S2). These results agree with those obtained in HT-treated *P. alba* × *Populus tremula* var. *glandulosa*^7^ as well as the reduced ABA levels in the roots and leaves of *P. simonii* under HW vs AW conditions (see below). Similar to the HT-induced differential expression of aquaporin genes, drought repressed the mRNA levels of *PIP 2;2* and three *TIP*s under AT whereas the transcript levels of these genes were up-regulated under HT in the roots. Drought decreased the transcript levels of *PIP2;2* and *TIP1;3* under AT but increased the mRNA levels of these genes under HT in the leaves of *P. simonii*. Aquaporins facilitate water transport across the membrane system^54^, therefore our results indicate that the aforementioned aquaporins probably play roles in water transport in the roots and leaves of poplars during the response to HT and/or drought.

### Overlapping heat-/drought-responsive genes are co-expressed and form hierarchical genetic regulatory networks

**Figure 3:**
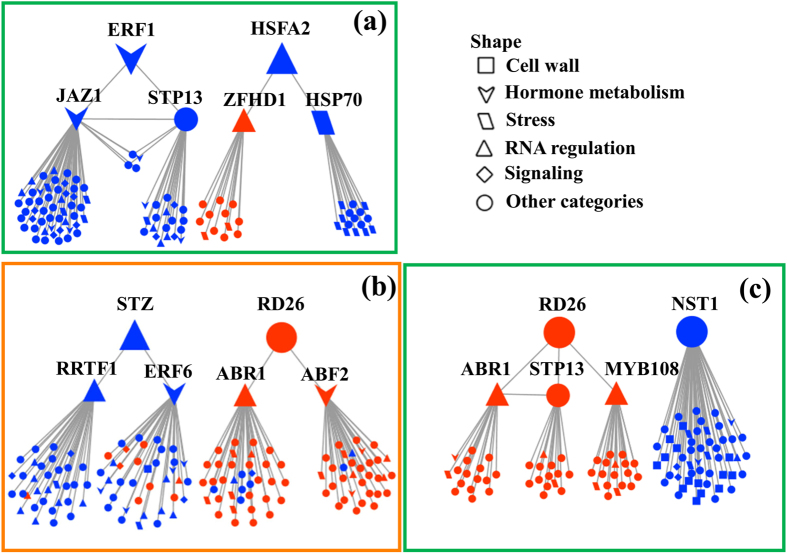
Hierarchical genetic networks for heat-responsive genes in the leaves (**a**) and hub genes (≥10 edges in Fig. S3a for the roots and ≥20 edges in Fig. S3b for the leaves) in the roots (**b**) and leaves (**c**) in response to drought. Gene to gene relationships were obtained by Popgenie v3.0 (http://popgenie.org/) using the exNet tool with a display threshold of 5 and an expand threshold of 7. Some hub genes are named in the networks and the abbreviations for these hub genes are as follows: *ERF1* (Potri.010G072300), *ETHYLENE RESPONSE FACTOR 1; HSPA2* (Potri.006G226800), *HEAT SHOCK TRANSCRIPTION FACTOR A2; JAZ1* (Potri.001G166200), *JASMONATE-ZIM-DOMAIN PROTEIN 1; STP13* (Potri.010G089800), *SUGAR TRANSPORT PROTEIN 13; ZFHD1* (Potri.010G169400), *ZINC FINGER HOMEODOMAIN 1; HSP70* (Potri.010G206600), *HEAT SHOCK PROTEIN 70; STZ* (Potri.002G119300), *SALT TOLERANT ZINC FINGER; RD26* (Potri.011G123300), *RESPONSIVE TO DESICCATION 26; RRTF1* (Potri.004G141200), *REDOX RESPONSIVE TRANSCRIPTION FACTOR 1; ERF6* (Potri.001G154200), *ETHYLENE RESPONSIVE ELEMENT BINDING FACTOR 6; ABR1* (Potri.002G065600), *ABA REPRESSOR1; ABF2* (Potri.002G125400), *ABA RESPONSIVE ELEMENTS-BINDING FACTOR 2; NST1* (Potri.011G153300)*, NAC SECONDARY WALL THICKENING PROMOTING FACTOR1; MYB108 (Potri.010G149900), MYB DOMAIN PROTEIN 108*. Detailed information about the differentially expressed genes in each network is presented in Supplementary Table S5. GO terms enriched for each hub gene in the sub-network are also presented in Supplementary Table S6.

Overall, our results suggest that HT and/or drought can induce tightly regulated gene networks related to stress responses and metabolic reprogramming in the roots and leaves of *P. simonii*. To corroborate this hypothesis, co-expression analysis was conducted using the overlapping heat-/drought-responsive genes in the roots and leaves of *P. simonii* (Figs 3 and S2, Table S5). Only 10 overlapping heat-responsive genes were found in poplar roots, so no co-expression was detected among these genes (data not shown). In the leaves, 76 genes (approximately 27%) among 285 query genes formed a heat-responsive hierarchical genetic network connected via 145 edges (Fig. 3a, Table S5). *ERF1* and *HSFA2* were located at the top of this hierarchical network, which controlled four subnetworks via *JAZ1, STP13, ZFHD1* and *HSP70*, thereby demonstrating that *ERF1* and *HSFA2* played key roles in co-regulating the transcript levels of overlapping heat-responsive genes in poplar leaves under both watering regimes (Fig. 3a). For the overlapping drought-responsive genes in the roots, 780 genes (approximately 64%) among 1221 query genes formed a drought-responsive co-expression network connected via 2532 edges (Fig. S2a, Table S5). Similarly, 1438 genes (approximately 78%) among 1838 query genes in the leaves formed a drought-responsive co-expression network connected via 6274 edges (Fig. S2b, Table S5). To identify the highly interconnected genes (hub genes) in the co-expression networks of *P. simonii* roots and leaves when exposed to drought, we defined each gene with more than 10 edges in the roots or more than 20 edges in the leaves as a hub gene. Based on these criteria, we identified 146 hub genes in the co-expression network for the roots (Fig. 3b) and 105 in the network for the leaves (Fig. 3c) that formed hierarchical genetic networks, in drought-exposed *P. simonii* (Table S5). Functional category analysis showed that the hub genes were enriched mostly in the RNA regulation and hormone metabolism categories in the dehydrated roots (28 of 146 hub genes, ca. 19%) and leaves (eight of 105 hub genes, ca. 8%) of poplars (Table S5). In particular, these hub genes included transcriptional up-regulation of *RD26* and *ABR1*, and down-regulation of *STZ* and *RRTF1* (Fig. 3b, Table S5). Importantly, the transcript levels of two hub genes involved in hormone metabolism, i.e., *ERF6* and *ABF2*, were decreased in drought-treated poplar roots (Fig. 3b, Table S5). Furthermore, *STZ* and *RD26* were located at the top of the hierarchical genetic network (Fig. 3b), thereby suggesting that they play key roles in coordinating gene co-expression in poplar roots during the response to drought. In drought-exposed poplar leaves, the hub genes assigned to the RNA regulation category included *RD26, ABR1, MYB108* and *NST1* (Fig. 3c, Table S5). Among these TFs, *RD26* and *NST1* were both located at the top of two subnetworks in the hierarchical genetic network of the hub genes (Fig. 3c).

To further explore the functions of hub genes involved in the hierarchical genetic regulatory networks in *P. simonii* roots and leaves in response to HT and/or water deficit (Fig. 3), we performed a GO term enrichment analysis (Table S6). The GO term analysis indicated that 34 GO terms in the roots and 24 GO terms in the leaves were significantly enriched in relation to the hub genes determined from the overlapping drought-responsive genes (Fig. 3, Table S6). In drought-treated roots, the four most significantly enriched GO terms comprised polysaccharide metabolic process (GO:0005976), metal ion binding (GO:0046872), peptidase inhibitor activity (GO:0030414) and endopeptidase inhibitor activity (GO:0004866), which were related to the hub genes *RRTF1, ERF6, ABR1* and *ABF2*, respectively (Fig. 3b, Table S6). In dehydrated poplar leaves, the most significantly enriched GO term was cellulose synthase (UDP-forming) activity (GO:0016760), which was associated with the hub gene *NST1* (Fig. 3c, Table S6).

Our data clearly demonstrate that transcriptomic reprogramming is co-ordinated in the roots and leaves of *P. simonii* in response to HT and/or drought. In contrast to the percentage of co-expressed genes under HT, the higher percentages of co-regulated genes in the roots and leaves of desiccated *P. simonii* suggest that withholding watering probably produces a more stressful environment for poplar roots and leaves than HT. Moreover, gene co-expression has been reported in *A. thaliana* when treated with heat stress and/or several other biotic and abiotic stressors^17^. Under drought conditions, co-regulation networks for transcriptomes have also been identified in herbaceous and woody plants such as rice^55^, the common sunflower^56^ and *Quercus suber*^57^. However, no previous studies have addressed the co-expression of genes in response to HT combined with drought in woody plants, and thus it is the first study of this issue. Moreover, GO term analysis indicated that hub genes were enriched for fundamental processes, including nitrogen compound metabolic process and RNA metabolic process. This suggests that the hub genes in each co-expression network play key roles in the cross-talk among different biological processes, thereby orchestrating transcriptomic reconfiguration in the roots and leaves of *P. simonii* in response to HT and/or desiccation.

### Physiological changes are linked to HT-/drought-induced transcriptomic reprogramming in poplar roots and leaves

**Figure 4:**
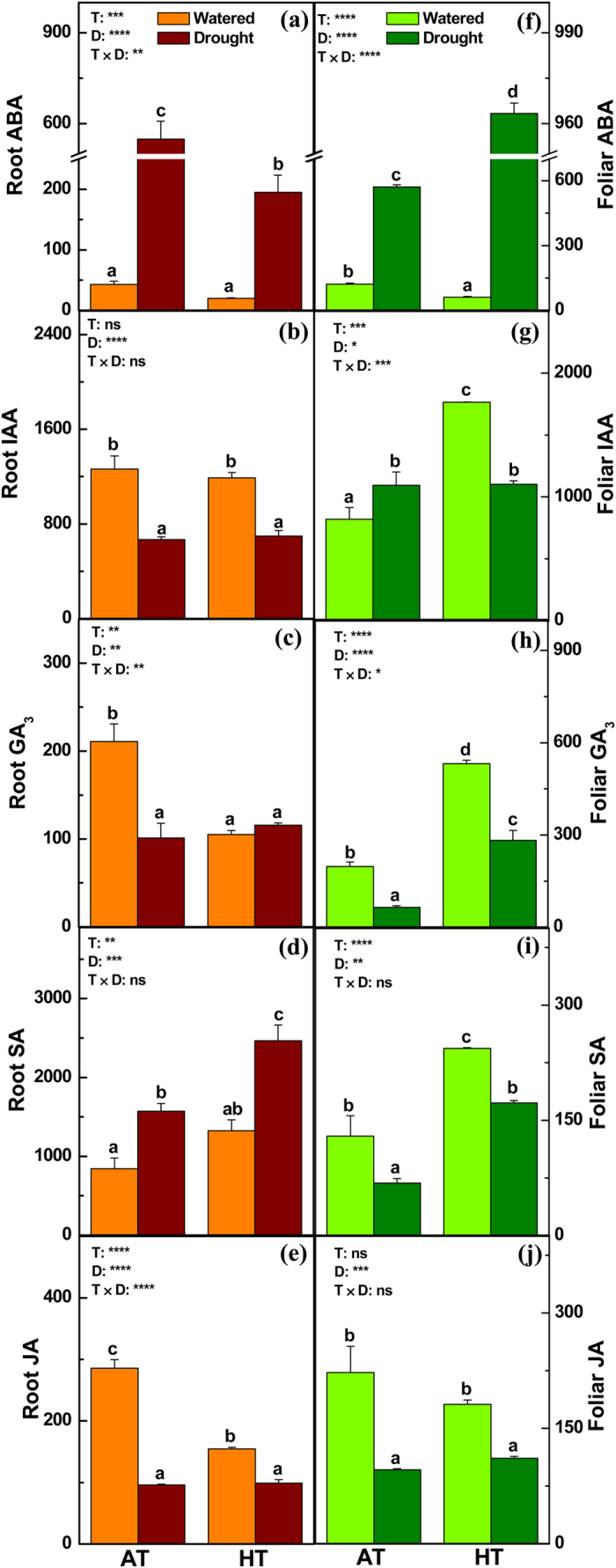
Concentrations (ng g^−1^ DW) of phytohormones in the roots (**a**–**e**) and leaves (**f**–**j**) of *P. simonii* treated at either ambient (A) or high (H) temperatures combined with one of two watering regimes (well-watered (W) or drought (D)). Bars indicate means ± SE (n = 6). Different letters on the bars indicate significant differences. *P*-values according to analysis of variance (ANOVA) tests for temperature (T), drought (D), and their interaction (T × D) are indicated. *P < 0.05; **P < 0.01; ***P < 0.001; ****P < 0.0001; ns: not significant.

**Figure 5:**
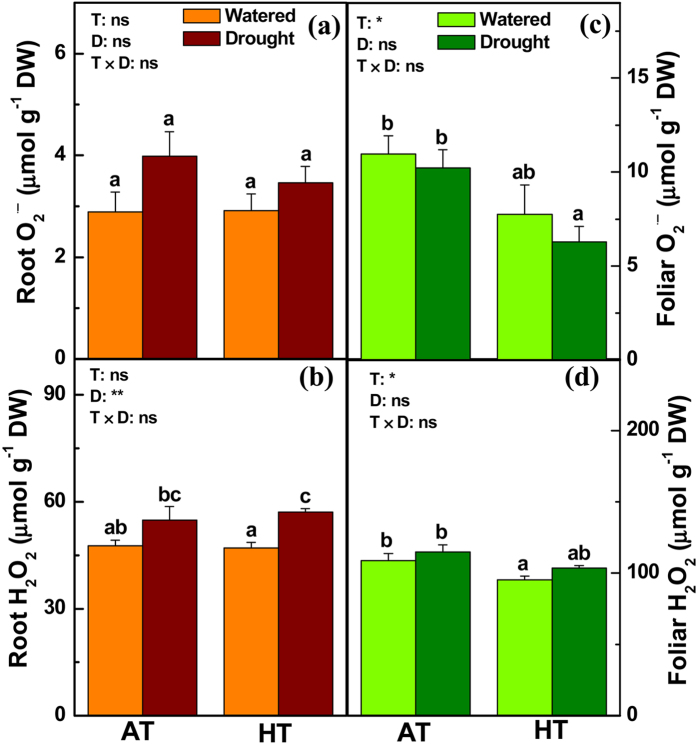
Concentrations of O_2_^⋅−^ and H_2_O_2_ in the roots (**a**,**b**) and leaves (**c**,**d**) of *P. simonii* treated at either ambient (A) or high (H) temperatures combined with one of two watering regimes (well-watered (W) or drought (D)). Bars indicate means ± SE (n = 6). Different letters on the bars indicate significant differences. *P*-values according to analysis of variance (ANOVA) tests for temperature (T), drought (D), and their interaction (T × D) are indicated. *P < 0.05; **P < 0.01; ns: not significant.

**Figure 6:**
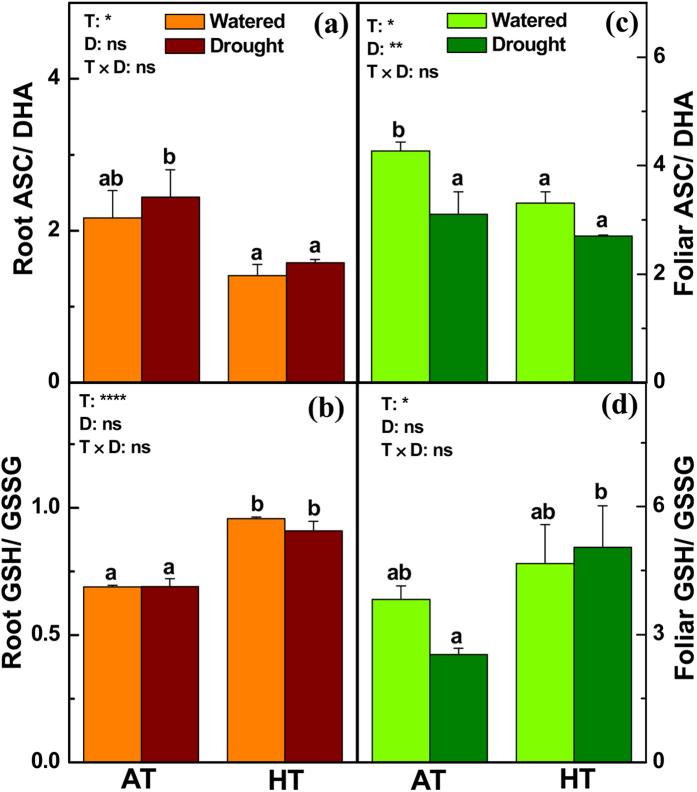
Ratio of ascorbate (ASC) relative to dehydroascorbate (DHA), and of glutathione (GSH) relative to oxidized GSH (GSSG) in the roots (**a**,**b**) and leaves (**c**,**d**) of *P. simonii* treated at either ambient (A) or high (H) temperatures combined with one of two watering regimes (well-watered (W) or drought (D)). Bars indicate means ± SE (n = 6). Different letters on the bars indicate significant differences. *P*-values according to the analysis of variance (ANOVA) tests for temperature (T), drought (D), and their interaction (T × D) are indicated. *P < 0.05; **P < 0.01; ***P < 0.001; ****P < 0.0001; ns: not significant.

To determine the physiological processes that were probably affected by HT and/or drought, we analyzed the water status, photosynthesis, *δ*^*13*^*C, δ*^*15*^*N*, and *δ*^*18*^*O* levels, carbohydrates, phytohormones, ROS, and antioxidants in the roots and leaves of *P. simonii* (Figs 4, 5 and 6, S3–S6, Table 1, S7,S8). The leaf water potential decreased by ca 20–25% in *P. simonii* under HT, but it was reduced dramatically by ca three times (in terms of the absolute values) under drought conditions (Table S7). The CO_2_ assimilation rate remained stable in *P. simonii* under either AT or HT, but it was inhibited by ca 95% under drought conditions (Table S8). Stomatal conductance (*g*_*s*_) was stable at HT under well-watered conditions, but it decreased more rapidly at HT than AT under drought conditions (Fig. S3). Moreover, *g*_*s*_ was reduced under drought conditions (Fig. S3). The greater photosynthetic inhibition in poplar leaves under drought compared with that under HT agreed with the decreased transcript levels of a large number of genes associated with photosynthesis under water deficit compared with HT (Table S5). *δ*^*13*^*C* was elevated in the roots of HT-treated *P. simonii*, whereas *δ*^*18*^*O* was reduced (Fig. S4). Similarly, in poplar roots exposed to drought, *δ*^*13*^*C* increased at both temperatures, while *δ*^*15*^*N* and *δ*^*18*^*O* increased under AT but decreased under HT (Fig. S4a–c). In poplar leaves, *δ*^*15*^*N* decreased under HT, whereas *δ*^*13*^*C* and *δ*^*18*^*O* were unaffected under HT (Fig. S4d–f). Interestingly, *δ*^*13*^*C, δ*^*15*^*N*, and *δ*^*18*^*O* were all elevated in the dehydrated leaves of *P. simonii* (Fig. S4d–f).

In the roots of *P. simonii*, the concentrations of total non-structural carbohydrates (TNCs) including glucose and sucrose were elevated under HT (Table 1). The TNC levels were also significantly higher in the roots exposed to drought than well-watered conditions, and the concentrations of glucose, inositol, and mannitol exhibited similar changes (Table 1). In agreement, a number of genes involved with major and minor carbohydrate metabolism, such as *starch branching enzyme 2.1 (SBE2.1*), *SBE2.2, sucrose-phosphatase 1 (SPP1*), *beta-fructofuranosidase 4 (BETAFRUCT4*), and *MYO-INOSITOL-1-PHOSTPATE SYNTHASE 3 (MIPS3*), were activated in drought-exposed roots (Table S5). In poplar leaves, the concentrations of TNCs including glucose, sucrose, and inositol, were significantly higher under HT than AT (Table 1). Similarly, the levels of TNCs, including sucrose, galactose, inositol, and starch, were elevated in the leaves exposed to drought (Table 1). In line with these results, a few genes involved with carbohydrate metabolism, including *SUS3, SUS6, ALPHA-AMYLASE-LIKE 2 (AMY2*), and *galactinol synthase 1 (GolS1*), were up-regulated in poplar leaves in response to HT and/or drought (Table S5).

Photosynthesis is closely related to water and carbon (C) metabolism in plants. Previous studies have shown that plant leaves often need to increase *g*_*s*_ to decrease the leaf temperature at HT^58^,^59^. However, a greater *g*_*s*_ can cause water losses from plants, which is unfavourable for plant survival under HT. Thus, plants must balance the leaf temperature and water losses. In well-watered *P. simonii* leaves, the stable water status, CO_2_ assimilation rate, and *g*_*s*_ under HT suggest that the poplars employed a conservative strategy to assimilate C and save water under HT, thereby resulting in unaltered *δ*^*13*^*C* and *δ*^*18*^*O* levels in the HT-exposed leaves. The CO_2_ assimilation rate remained unaltered in HT-treated poplar leaves, but elevated concentrations of TNCs, including glucose, sucrose, galactose, and inositol, were detected in the leaves when exposed to HT, probably due to the reduced demand for the soluble sugars and sugar alcohols used as structural components, such as cellulose, hemicellulose, and lignin, in HT-exposed poplar leaves. Similarly, elevated concentrations of carbohydrates have also been reported in HT-treated woody plants^7^,^60^. In contrast to the small HT-induced changes in water status and C metabolism, water deprivation led to greater alterations in the water status, CO_2_ assimilation rate and carbohydrates levels in *P. simonii*, thereby suggesting that drought had greater effects on physiological processes than HT. Moreover, the drought-induced accumulation of TNCs, including glucose, sucrose, galactose, and inositol, is consistent with the results of other studies^7^,^61^,^62^, and thus this probably contributes to osmotic adjustment to combat water losses by the roots and leaves in *P. simonii. MIPS3* encodes myo-inositol-1-phosphate synthase isoform 3, which is required for myo-inositol biosynthesis in plants under stress conditions^63^. *SUS6* encodes a protein with a sucrose synthase activity (SUS6), which is involved with sucrose biosynthesis in plants^64^. The up-regulated mRNA levels of these genes agreed with the elevated concentrations of inositol in the roots and sucrose in the leaves of drought-treated *P. simonii*.

In the roots of *P. simonii*, HT triggered lower ABA concentrations under drought conditions, and decreased the gibberellin (GA_3_) and JA levels under well-watered conditions, but higher SA levels under both watering regimes (Fig. 4a–e). In the roots, desiccation led to significant increases in the concentrations of ABA and SA, but lower levels of IAA, GA_3_, and JA (Fig. 4a–e). Accordingly, the mRNA levels of a few genes involved in ABA signaling pathways, such as *NCED3, ABRE BINDING FACTOR 4 (ABF4*), and *a zeaxanthin epoxidase*, were increased, whereas the transcript levels of a number of genes involved in the auxin, gibberellin, and jasmonate signalling cascades, including *GH3.9, small auxin up-regulated 71 (SAUR71*), and *JASMONATE RESISTANT 1 (JAR1*), were decreased in drought-exposed poplar roots (Table S5). In poplar leaves, the concentrations of ABA, IAA, GA_3_, and SA were significantly higher under HT than AT (Fig. 4f–i). The foliar ABA levels were elevated due to water deficit, but the foliar levels of IAA under HT, as well as GA_3_, SA, and JA under both temperatures, were decreased under drought conditions (Fig. 4f–j). Consistently, the mRNA levels of a number of genes related to ABA signalling cascades, such as *ABA-responsive protein-related, NCED3*, and *ABF3*, were elevated, whereas the transcript levels of genes involved in IAA, GA, SA and JA signalling pathways, including *SAUR51, GH3.10, GIBBERELLIN 3-OXIDASE 4 (GA3OX4*), and *UDP-glucoronosyl/UDP-glucosyl transferase 74F1 (UGT74F1*), were decreased in poplar leaves treated with HT and/or drought (Table S5).

Plant hormones play essential roles in stress perception and modulating the physiological responses to HT and/or drought^6^,^7^,^65^. Changes in the concentrations of phytohormones often occur in plants in response to HT and/or water deficit, which can trigger signal transduction in plant cells^6^,^7^,^65^. ABA accumulation occurs frequently in plants exposed to HT and/or desiccation^6^,^7^,^65^. Indeed, foliar ABA elevation in *P. simonii* treated with HT and/or drought can lead to stomatal closure and decreased CO_2_ assimilation. In addition, IAA plays a role in the molecular and physiological reconfiguration of plants in response to HT and/or drought^7^,^48^. Decreased IAA concentrations have been reported in herbaceous plants and poplars in response to HT and/or water deficit^6^,^7^. The lower IAA levels found in the roots and leaves of dehydrated *P. simonii* are consistent with previous findings in drought-exposed *P. alba* × *Populus tremula* var. *glandulosa*^7^. Other phytohormones, such as GAs, SA, and JA, are also involved in the plant signalling networks related to responses to HT and/or drought^6^,^7^,^65^. The accumulated foliar GA_3_ levels and decreased JA levels in the roots and leaves of HT-treated *P. simonii* are consistent with the results found in HT-treated *P. alba* × *Populus tremula* var. *glandulosa*^7^. Similarly, the drought-induced decreases in the GA_3_ and JA levels in the roots and leaves of *P. simonii* are also in agreement with the phytohomornal changes reported previously in desiccation-exposed poplars^7^. The changes in the levels of these hormones in the roots and leaves of *P. simonii* suggest that they also participated in signalling pathways that responded to HT and/or water deprivation. Plant hormones are important for growth, development and stress responses, so phytohormone metabolism and signalling are strictly controlled at the transcriptional level in plants. For instance, *NCED3* encodes 9-cis-epoxycarotenoid dioxygenase which is a key enzyme for the biosynthesis of ABA in plants^66^. PP2C proteins are negative regulators of ABA signaling^67^. At the transcriptional regulation level, *PP2Cs* are often up-regulated due to negative feedback in plants in response to increased ABA levels and/or drought^61^. Similarly, PYR1-like (PYL) proteins are ABA receptors^67^. The transcript levels of *PYL*s are often decreased in plants in response to ABA accumulation and/or drought exposure^67^. The elevated mRNA levels of *NCED3* and *PP2C*, as well as the reduced transcript levels of *PYL*s in the roots and/or leaves of drought-exposed *P. simonii* agree well with ABA accumulation responses in poplar tissues in the present study and in other poplars treated by desiccation^7^. *GH3.9* encodes a member of the GH3 family auxin-responsive proteins^68^. The decreased *GH3.9* transcript levels in the roots of drought-exposed *P. simonii* agree with the reduced levels of IAA in these poplar tissues.

In the roots of *P. simonii*, HT had no effects on the levels of O_2_^⋅−^ and H_2_O_2_, but drought increased the H_2_O_2_ concentrations (Fig. 5a,b). In the roots, HT resulted in lower concentrations of ascorbate (ASC) and oxidized-glutathione (GSSG), unaltered dehydroascorbate (DHA) levels, but higher levels of reduced-glutathione (GSH) (Fig. S5). Drought decreased the ASC and DHA levels, whereas the concentrations of GSH and GSSG were unchanged (Fig. S5). Thus, HT reduced the ratio of ASC relative to DHA, but increased the ratio of GSH relative to GSSG in poplar roots (Fig. 6a,b). Water deficit had no influence on the ratio of ASC relative to DHA and the ratio of GSH relative to GSSG in the roots (Fig. 6a,b). HT inhibited the activities of antioxidant enzymes including ascorbate peroxidase (APX) and glutathione reductase (GR) in poplar roots, whereas drought had little effect on the activities of these enzymes (Fig. S6). In poplar leaves, the concentrations of O_2_^⋅−^ and H_2_O_2_ were slightly lower under HT than AT, and the foliar ROS levels were unaffected by water deficit (Fig. 5c,d). The foliar concentrations of ASC and DHA increased, whereas the foliar GSSG levels decreased under HT compared with AT (Fig. S5). The foliar levels of ASC and DHA were decreased under drought conditions, whereas the foliar concentrations of GSH and GSSG were elevated due to the water deficit (Fig. S5). Thus, the foliar ratio of ASC relative to DHA was lower under HT than AT, whereas the foliar ratio of GSH relative to GSSG was higher under HT than AT (Fig. 6c,d). Moreover, the foliar ratio of ASC relative to DHA was decreased by drought (Fig. 6c). In the leaves, the activities of superoxide dismutase (SOD) and APX were inhibited under HT, whereas the SOD activities were induced under desiccation conditions (Fig. S6). In agreement with these results, several genes related to oxidative stress and detoxification process were significantly differentially expressed in poplar roots and/or leaves exposed to HT and/or drought (Table S5). For instance, the transcript levels of genes including *monodehydroascorbate reductase 1 (MDAR1*), *GLUTATHIONE PEROXIDASE 4 (GPX4*), *GPX6*, and *GLUTATHIONE SYNTHETASE 2 (GSH2*), which encode proteins involved with the glutathione-ascorbate cycle, were increased in the roots and/or leaves of drought-treated *P. simonii* (Table S5). The mRNA levels of *ASCORBATE PEROXIDASE 4 (APX4*) decreased in poplar leaves exposed to drought (Table S5).

HT and drought are abiotic stresses, which alone or combined can modify the homeostasis between ROS and antioxidants in plants. In *P. alba* × *P. tremula* var. *glandulosa* saplings, HT induces lower root O_2_^⋅−^ levels, but drought triggers the overproduction of O_2_^⋅−^ and H_2_O_2_^7^. The ROS changes detected in the current study are consistent with those found in *P. alba* × *P. tremula* var. *glandulosa*. Antioxidants such as ASC and GSH play important roles in scavenging the ROS that is overproduced in plants when exposed to biotic and abiotic stresses. It has been demonstrated that the ASC and GSH levels decrease, whereas the concentrations of their oxidative forms, i.e., DHA and GSSG, increase during the scavenging of ROS overproduced in woody plants treated with various stresses^69^. In the current study, the lower HT-induced ratio of ASC relative to DHA may be ascribed to the reductions in ASC and the increases in DHA in *P. simonii* under HT, which were probably also associated with ASC conversion into DHA during ROS scavenging. Similarly, the higher HT-triggered ratio of GSH relative to GSSG was due to the increases in GSH and decreases in GSSG in the roots and leaves of *P. simonii* treated with HT, possibly with limited impacts on ROS scavenging. In addition to non-enzymatic antioxidants, antioxidative enzymes play roles in scavenging the extra ROS produced in woody plants when exposed to HT and/or desiccation^7^. However, few antioxidative enzymes exhibited changes in their activities in *P. simonii* in response to HT and/or drought, thereby suggesting that enzymatic antioxidants had limited effects on surplus ROS scavenging in HT- and/or drought-treated *P. simonii*. The shifted in the balance between ROS and antioxidants in *P. simonii* exposed to HT and/or desiccation was also associated with the underlying transcriptional regulation of genes that are involved in the metabolism of ROS and antioxidants. *GPX4* and *GPX6* encode two glutathione peroxidase family members which catalyze reduced GSH and hydrogen peroxide to produce GSSG and water, thereby leading to the detoxification of H_2_O_2_^70^. The increased mRNA levels of *GPX4* and *GPX6* agreed with the elevated H_2_O_2_ and decreased GSH levels in *P. simonii* roots treated with water deficit. Glutathione synthetases catalyze the condensation of gamma-glutamylcysteine and glycine to form glutathione^71^. Thus, the elevated mRNA levels of *GLUTATHIONE SYNTHETASE 2 (GSH2*) were consistent with the foliar accumulation of GSH in drought-treated *P. simonii*. APXs are enzymes that detoxify peroxides such as hydrogen peroxide by using ascorbate to produce dehydroascorbate and water^72^. The down-regulated transcript levels of *APX4* agreed with the decreased concentrations of ASC and DHA in the leaves of *P. simonii* exposed to drought.

In conclusion, RNA-sequencing analysis showed that a large number of genes were differentially expressed in the roots and leaves of *P. simonii* in response to HT and/or desiccation, but only a small number of these genes were identified as overlapping heat-/drought-responsive genes, which were mainly involved in RNA regulation, transport, hormone metabolism, and stress categories. Furthermore, the overlapping heat-/drought-responsive genes were co-expressed and formed hierarchical genetic regulatory networks, which suggests that these genes played key roles in coordinating transcriptomic reprogramming in the roots and leaves of poplars during the response to HT and/or drought. HT-/drought-induced transcriptomic reprogramming was linked to physiological responses in the poplar roots and leaves. The HT- and/or drought-triggered accumulation of TNCs, including glucose, sucrose, and inositol, in poplar roots and/or leaves corresponded well with the overexpression of several genes, including *MIPS3, SUS3*, and *SUS6*, which are involved with TNC metabolism. Similarly, the HT- and/or drought-induced accumulation of ABA and decreases in the levels of IAA and other phytohormones were in agreement with the up-regulated transcript levels of a few genes, such as *NCED3, ABF3*, and *PP2C*, as well as the reduced mRNA levels of other genes, including *GH3.9, GH3.10*, and *JAR1*. Moreover, HT and/or drought shifted the homeostasis between ROS and antioxidants, which was also linked with the differential expression of genes involved with the metabolism of ROS and antioxidants in poplar roots and leaves. These results suggest that overlapping heat-/drought-responsive genes may play key roles in the transcriptional and physiological reconfiguration of poplar roots and leaves in response to HT and/or drought under future climatic scenarios.

## Methods

### Plant cultivation and treatments

Plant cultivation and treatments were performed in a similar manner to that described previously^7^. Briefly, *Populus simonii* cuttings were rooted and subsequently planted in plastic pots filled with soil. Plants were cultivated in a glasshouse for 3 months before they were assigned to six climate chambers, with 18 plants in each chamber (day/night temperature, 25/20 °C; light/dark, 16/8 h; light intensity, 250 μmol m^−2^ s^−1^ at plant height; relative humidity, 60%). Before the experimental treatment, plants were grown for 2 weeks with daily irrigation to field capacity and each plant was supplied with 50 mL Hoagland nutrient solution every 2 days.

In the temperature treatments, three climate chambers were assigned to HT and the other three were set as AT. The day/night temperatures of the chambers treated with HT and AT were set as 30 ± 0.8/25 ± 0.6 °C and 25 ± 0.4/20 ± 0.2 °C, respectively. For the drought treatments, 12 plants with similar growth performance in each chamber were divided into two groups (6 plants in each group). The plants in each group were treated using one of two watering regimes (well-watered (W) by watering the soil up to 80% of the field capacity and drought (D) by withholding watering). The temperature and drought treatments were initiated on the same day.

### Gas exchange and predawn leaf water potential (LWP)

During the treatments, three plants from each treatment in each chamber were selected for daily monitoring of *g*_*s*_. Three mature leaves (leaf plastochron index, LPI = 7–9) were analyzed from each selected plant, as described previously^61^. Before harvesting, gas exchange and the respiration rates (*R*_*s*_) of the leaves were measured as described previously^7^.

The leaves (LPI = 8) selected for gas exchange measurements were used to determine the predawn LWP. LWP was measured after daily predawn irrigation on the day of harvest using a Scholander pressure chamber (model 600; PMS, Albany, OR, USA), as described previously^73^.

### Harvesting and relative water contents

The experimental treatments were kept for 8 days until *g*_*s*_ reached zero in the plants exposed to AT and drought. Subsequently, six plants were harvested from each treatment in each chamber. The maximum root length, leaf number, and fresh weights of the roots and leaves were recorded for each plant during the harvest (Table S10). The harvested roots and leaves from each plant were separated before wrapping with tinfoil and freezing immediately in liquid nitrogen. The frozen samples were ground into a fine powder in precooled jars (liquid nitrogen) in a ball mill (MM400, Retsch, Haan, Germany) and stored at −80 °C until their further analysis. Within the same treatment, for each tissue, equal amounts of powder from each of three plants (each plant came from one of three AT or HT chambers) were pooled, thereby resulting in six pooled samples for each tissue and treatment which were used for further biochemical and molecular analyses. Aliquots of powdered root or leaf samples (ca 80 mg) were dried at 60 °C for 72 h to determine the ratios of fresh relative to dry biomass and the relative water contents (RWC), which were calculated as follows: RWC% = (Fresh weight – Dry weight)/Fresh weight × 100.

### RNA isolation and sequencing

RNA isolation and sequencing of poplar roots and leaves were performed as described previously^74^. Briefly, total RNA was isolated from the pooled root or leaf powder as described above using a plant RNA extraction kit (R6827, Omega Bio-Tek, GA, USA), i.e., six total RNA samples were obtained for each tissue within each treatment. Genomic DNA in the RNA extract was digested using DNase I (E1091, Omega Bio-Tek, GA, USA). Next, equal amounts of total RNA from each preparation of each tissue within each treatment were pooled for subsequent library construction and RNA sequencing, i.e., one library was established per tissue for each treatment and used for RNA sequencing. Library construction and Illumina sequencing were performed by Shanghai Biotechnology Corporation (Shanghai, China). The libraries obtained for the roots and leaves were sequenced by using Illumina Genome Analyzer HiSeq 2500 and HiSeq 2000 systems, respectively, thereby obtaining single-end reads measuring 50 bp length, which exhibited the visible difference in raw reads between the roots and leaves (Table S1). High quality reads that passed the Illumina quality filters were selected for further sequence analysis. The sequencing datasets are available at NCBI Sequence Read Archive (SRA, http://www.ncbi.nlm.nih.gov/Traces/sra/, accession number: SRP064930).

### Sequence analysis

The sequence analysis was performed in a similar manner to that described in a previous study^74^. Briefly, high quality reads were mapped to the mRNA reference sequence of *P. trichocarpa* (ftp://ftp.jgipsf.org/pub/compgen/phytozome/v9.0/Ptrichocarpa/assembly/Ptrichocarpa_210.fa.gz) using the spliced mapping algorithm in tophat (version:2.0.9)^75^, where the settings allowed two mismatches and multihits ≤10. The gene expression levels were calculated using the fragments per kilobase of exon model per million mapped reads (FPKM) method in cufflink (version 2.1.1)^76^. The differential expression of genes was calculated based on the normalized FPKM using the DEGseq package^77^. The *P*-values of statistics were adjusted using the method described by Benjamini and Hochberg^78^ and the false discovery rate (FDR) was used to determine the threshold *P*-value in multiple test analyses. The FPKM values obtained by applying these criteria to *P. simonii* roots and leaves exposed to either AT or HT combined with one of two watering regimes (well-watered (W) or drought (D)) were used in further analyses. The fold changes in differentially expressed genes were calculated by comparing the FPKM values for HW vs AW, HD vs AD, AD vs AW, and HD vs HW in the roots or leaves of *P. simonii*. Significantly differentially expressed genes in *P. simonii* roots and leaves were determined based on FDR values <0.05 and fold changes ≥2 (or less than −2).

### Annotation, functional categorization, co-expression analysis and gene ontology

Significantly differentially expressed genes were annotated as described previously^79^ with minor modifications. In brief, the coding sequences of significantly differentially expressed genes were retrieved from the *P. trichocarpa* database (version 3.0). The closest *Arabidopsis (A. thaliana*) homologue (AGI identification) of a *P. simonii* gene was determined by translated nucleotide BLAST (BLASTX) analysis of the coding sequence for the best *P. trichocarpa* hit against the *Arabidopsis* protein sequence dataset. The annotations were taken from the latest release of The *Arabidopsis* Information Resource genome database (TAIR10).

To identify the overlapping heat-responsive genes, common poplar gene IDs were searched among significantly differentially expressed genes under both conditions (HW vs AW, HD vs AD) in the roots or leaves of *P. simonii*. Similarly, the overlapping drought-responsive genes were identified among the significantly differentially expressed genes under both conditions (AD vs AW, HD vs HW). Subsequently, the gene IDs of the overlapping heat-/drought-responsive genes in poplar roots and leaves were submitted to MapMan for functional category analysis, as suggested previously^80^. The overlapping heat- or drought-responsive genes with functional categories assigned by MapMan were used for co-expression analysis, as described by Sjödin^81^ with the modifications suggested by Sundell^82^, using an online open resource (http://popgenie.org/). The co-expression analysis results were visualized in Cytoscape version 3.2.1 as described previously^83^. Hub genes were defined based on the edges assigned to genes in the co-expression network. Hierarchical genetic networks were also constructed according to the regulatory roles of TFs in the co-expression networks.

To identify significantly enriched GO terms in each regulatory module of the hierarchical genetic networks obtained for *P. simonii* roots and leaves, the poplar gene IDs of all the genes in each module were used for singular enrichment analysis using the agriGO database (http://bioinfo.cau.edu.cn/agriGO/index.php), as suggested previously^84^. Fisher method and Yekutieli (FDR under dependency) were used for statistical test and Multi-test adjustment, respectively. *P*-value threshold for significance was 0.05 and the minimum number of mapping entries was 5 for complete GO analysis.

### Validation of RNA-sequencing data by RT-qPCR

To validate the RNA-sequencing analysis data, we performed RT-qPCR using total RNA and gene specific primers (Table S9) according to the method described by Li^85^. For each tissue within each treatment, six samples of total RNA which had been pooled for RNA sequencing were used for PCR. The PCR products were sequenced and aligned with homologues from other model plants to ensure their validity (Fig. S7). *Actin2/7* and 18 S ribosomal RNA were used as reference genes. The gene expression correlations were compared between the RNA sequencing data and the RT-qPCR results.

### Determination of soluble sugars, sugar alcohols, starch and stable isotope compositions

Soluble sugars and sugar alcohols were analyzed using a GC-MS system (Thermo Electron Corporation, Austin, TX), as described previously^86^. The starch concentrations in fine roots and leaves were analyzed using the anthrone method, as described previously^86^. Absorption was determined spectrophotometrically at 620 nm. A standard curve was established using serially diluted solutions of glucose and the starch concentrations were expressed as glucose equivalents. Stable carbon, nitrogen, and oxygen isotope compositions were also determined in the poplar roots and leaves (Methods S1).

### Plant hormone concentrations

The concentrations of ABA, IAA, GA_3_, SA, and JA in the root and leaf samples from *P. simonii* were determined by high performance liquid chromatography (LC-20AT, Shimadzu, Kinh Do, Japan)–electrospray tandem mass spectrometry (API 2000TM, Allen-Bradley Milwaukee, USA), according to a method described previously^87^.

### Determination of O_2_^⋅−^ and H_2_O_2_

The concentrations of the superoxide anion (O_2_^⋅−^) and H_2_O_2_ in root and leaf samples were determined spectrophotometrically at 530 and 410 nm, respectively, according to a published method^79^.

### Determination of non-enzymatic antioxidants and antioxidative enzyme activities

The concentrations of ASC, DHA, GSH and GSSG were analyzed in the root and leaf samples. ASC and DHA were determined based on a published protocol^88^ with minor modifications^89^. GSH and GSSG were determined according to a previously reported method^88^. Antioxidative enzyme activities were also analyzed in poplar roots and leaves (Methods S1).

### Statistical analysis

Statistical tests were performed with Statgraphics (STN, St Louis, MO, USA). The data were tested to confirm the normality of their distributions before statistical analyses. For the experimental variables, two-way ANOVAs were performed with temperature and drought as the two main factors. Differences between means were considered significant when *P* < 0.05 according to the ANOVA F-test. The Ct values obtained from qPCR were normalized and the relative fold changes in transcripts were calculated using the relative expression software tool, REST^90^.

## Additional Information

**How to cite this article:** Jia, J. *et al*. Comparative transcriptomic analysis reveals the roles of overlapping heat-/drought-responsive genes in poplars exposed to high temperature and drought. *Sci. Rep.* **7**, 43215; doi: 10.1038/srep43215 (2017).

**Publisher's note:** Springer Nature remains neutral with regard to jurisdictional claims in published maps and institutional affiliations.

## Supplementary Material

Supplementary Data MM V11

Supplementary Data Table S1-S6

## Figures and Tables

**Table 1:** Concentrations (nmol g^−1^ DW) of soluble sugars, sugar alcohols, starch, and total non-structural carbohydrates (TNCs) in the roots and leaves of *P. simonii* treated at either ambient (A) or high (H) temperatures combined with one of two watering regimes (well-watered (W) or drought (D).

Tissue	Treatment	Glucose (×10^3^)	Sucrose (×10^3^)	Galactose	Inositol (×10^3^)	Mannitol	Starch (×10^3^)	TNCs (×10^3^)
Roots	AW	6.9 ± 1.5 a	11.0 ± 0.9 b	45.9 ± 1.4 c	153.6 ± 13.7 a	134.5 ± 10.7 a	93.6 ± 10.4 b	265.2 ± 12.0 a
	AD	11.3 ± 2.5 a	5.9 ± 2.3 ab	10.3 ± 4.5 b	253.6 ± 65.3 a	345.2 ± 38.1 b	59.7 ± 10.5 a	330.9 ± 75.7 a
	HW	9.2 ± 1.3 a	1.0 ± 0.0 a	0.8 ± 0.1 a	221.8 ± 28.7 a	175.7 ± 1.7 a	82.2 ± 3.0 ab	314.3 ± 31.2 a
	HD	23.7 ± 1.4 b	32.6 ± 2.4 c	1.4 ± 0.2 a	562.2 ± 22.2 b	424.2 ± 49.3 b	71.4 ± 5.2 ab	690.3 ± 21.4 b
Leaves	AW	1.4 ± 0.1 a	3.7 ± 0.7 a	11.7 ± 0.8 a	51.4 ± 3.7 a	21.1 ± 0.8 a	30.9 ± 2.5 a	87.4 ± 6.0 a
	AD	1.5 ± 0.1 a	4.8 ± 0.1 a	16.3 ± 0.5 b	56.4 ± 5.8 a	25.3 ± 1.6 a	49.4 ± 2.2 b	112.1 ± 5.2 b
	HW	2.5 ± 0.1 b	3.2 ± 0.8 a	11.2 ± 0.9 a	63.0 ± 1.7 a	25.6 ± 1.3 a	24.4 ± 3.7 a	93.1 ± 5.8 a
	HD	2.6 ± 0.3 b	7.7 ± 0.6 b	20.2 ± 1.3 c	94.0 ± 2.0 b	23.8 ± 2.4 a	44.5 ± 1.7 b	148.8 ± 0.2 c
Roots	T	**	**	****	**	ns	ns	**
	D	***	***	***	***	***	*	***
	T × D	*	****	***	*	ns	ns	**
Leaves	T	***	*	ns	***	ns	ns	**
	D	ns	**	***	**	ns	***	****
	T × D	ns	*	*	**	ns	ns	*

Data indicate the mean ± SE (n = 6). Different letters after values in the same column for the same tissue indicate significant differences. *P*-values according to analysis of variance (ANOVA) tests for temperature (T), drought (D), and their interaction (T × D) are also indicated. *P < 0.05; **P < 0.01; ***P < 0.001; ****P < 0.0001; ns: not significant. TNCs were calculated as the sum of glucose, sucrose, galactose, inositol, mannitol, and starch.
