# Supplementary material for: Comparative transcriptomic analysis reveals the roles of overlapping heat-/drought-responsive genes in poplars exposed to high temperature and drought
Source: Sci Rep. 2017 Feb 24;7:43215. doi: 10.1038/srep43215 (PMC5324098; doi:10.1038/srep43215)
Supplement: Supplementary Data MM V11 [file srep43215-s1.doc]

**Supplementary Information to:**

**Comparative transcriptomic analysis reveals the roles of overlapping heat-/drought-responsive genes in poplars exposed to high temperature and drought**

Jingbo Jia, Jing Zhou, Wenguang Shi, Xu Cao, Jie Luo, Andrea Polle, Zhi-Bin Luo^*^

*Correspondence: **Zhi-Bin Luo**

E-mail: [luozbbill@163.com](mailto:luozbbill@163.com)

**Methods S1**

*Determination of stable carbon, nitrogen, and oxygen isotope compositions*

*δ^13^C* and *δ^15^N* in the root and leaf samples were analyzed using an elemental analyzer (NA 2500; CE Instruments, Rodano, Italy) and a mass spectrometer (Delta Plus; Finnigan MAT, Bremen, Germany) with an interface (Conflo III; Finnigan MAT), according to a published method with minor modifications . Additionally, *δ^18^O* was determined in the root and leaf samples according to the method with minor modifications of .

*Determination of activities of antioxidative enzymes*

Soluble proteins were extracted from the fresh samples and used to quantify the activities of antioxidative enzymes as reported previously . The enzyme activities of superoxide dismutase (SOD, EC 1.15.1.1), catalase (CAT, EC 1.11.1.6), ascorbate peroxidase (APX, EC 1.11.1.11), and glutathione reductase (GR, EC 1.8.1.7) were determined spectrophotometrically at 560 nm, 240 nm, 290 nm, and 340 nm, respectively, as described previously .

**References**

Cernusak, L.A., K. Winter and B.L. Turner. 2009. Physiological and isotopic (delta C-13 and delta O-18) responses of three tropical tree species to water and nutrient availability. Plant Cell and Environment. 32:1441-1455.

He, J., C. Ma, Y. Ma, H. Li, J. Kang, T. Liu, A. Polle, C. Peng and Z.B. Luo. 2013. Cadmium tolerance in six poplar species. Environ Sci Pollut Res Int. 20:163-74.

He, J., J. Qin, L. Long, Y. Ma, H. Li, K. Li, X. Jiang, T. Liu, A. Polle, Z. Liang and Z.B. Luo. 2011. Net cadmium flux and accumulation reveal tissue-specific oxidative stress and detoxification in Populus x canescens. Physiol Plant. 143:50-63.

Jia, J., S. Li, X. Cao, H. Li, W. Shi, A. Polle, T.X. Liu, C. Peng and Z.B. Luo. 2016. Physiological and transcriptional regulation in poplar roots and leaves during acclimation to high temperature and drought. Physiologia Plantarum. 157:38-53.

Luo, J., H. Li, T.X. Liu, A. Polle, C.H. Peng and Z.B. Luo. 2013. Nitrogen metabolism of two contrasting poplar species during acclimation to limiting nitrogen availability. Journal of Experimental Botany. 64:4207-4224.

Werner, R.A., B.A. Bruch and W.A. Brand. 1999. ConFlo III - An interface for high precision delta(13)C and delta(15)N analysis with an extended dynamic range. Rapid Communications in Mass Spectrometry. 13:1237-1241.

**Table S7** Predawn leaf water potential (LWP) and relative water contents (RWC) in the roots and leaves of *P. simonii* treated at either ambient (A) or high (H) temperatures combined with one of two watering regimes (well-watered (W) or drought (D)). Data indicate means ± SE (n = 6). Different letters after values in the same column indicate significant differences. *P*-values according to analysis of variance (ANOVA) tests for temperature (T), drought (D), and their interaction (T × D) are also indicated. *: *P* < 0.05; **: *P* < 0.01; ***: *P* < 0.001; ****: *P* < 0.0001; ns: not significant.

| Treatment | LWP (MPa) | Root RWC (%) | Foliar RWC (%) |
| --- | --- | --- | --- |
| AW | -0.48 ± 0.03 c | 86.0 ± 1.5 b | 78.9 ± 0.6 b |
| AD | -2.02 ± 0.01 b | 78.0 ± 1.5 a | 54.8 ± 3.0 a |
| HW | -0.60 ± 0.07 c | 86.3 ± 0.8 b | 77.4 ± 0.4 b |
| HD | -2.43 ± 0.12 a | 77.7 ± 0.6 a | 52.7 ± 0.3 a |
| T | ** | ns | ns |
| D | **** | *** | **** |
| T × D | ns | ns | ns |

**Table S8** The CO_2_ assimilation rate (***A***, μmol CO_2_ m^−2^ s^−1^), respiration rate (***R***, μmol CO_2_ m^−2^ s^−1^), transpiration rate (***E***, mmol H_2_O m^−2^ s^−1^), stomatal conductance (***g_s_***, mol H_2_O m^−2^ s^−1^) and the ratio of intercellular to ambient CO_2_ concentration (*C_i_*/*C_a_*) in the leaves of *P. simonii* treated at either ambient (A) or high (H) temperatures combined with one of two watering regimes (well-watered (W) or drought (D)). Data indicate means ± SE (n = 6). Different letters after values in the same column indicate significant differences. *P*-values according to analysis of variance (ANOVA) tests for temperature (T), drought (D), and their interaction (T × D) are also indicated. *: *P* < 0.05; **: *P* < 0.01; ***: *P* < 0.001; ****: *P* < 0.0001; ns: not significant.

| Treatment | *A* | *R* | *E* | *g_s_* | *C_i_/C_a_* |
| --- | --- | --- | --- | --- | --- |
| AW | 13.5 ± 0.4 b | 1.14 ± 0.16 b | 4.4 ± 0.1 b | 0.194 ± 0.011 b | 0.68 ± 0.01 a |
| AD | 0.6 ± 0.0 a | 0.13 ± 0.03 a | 0.3 ± 0.0 a | 0.009 ± 0.001 a | 0.84 ± 0.05 b |
| HW | 13.6 ± 0.6 b | 1.17 ± 0.13 b | 4.3 ± 0.5 b | 0.302 ± 0.010 b | 0.74 ± 0.02 b |
| HD | 0.3 ± 0.1 a | 0.05 ± 0.00 a | 0.2 ± 0.0 a | 0.006 ± 0.001 a | 1.25 ± 0.02 c |
| T | ns | ns | ns | ns | *** |
| D | **** | **** | **** | **** | **** |
| T × D | ns | ns | ns | ns | * |

**Table S9 Primers used for RT-qPCR.**

| **Gene symbol** | **Poplar gene model** | **Gene name** | **Closest AGI** | **Primer-Forward** | **Primer-Reverse** | **PCR efficiency (%)** |
| --- | --- | --- | --- | --- | --- | --- |
| *MIPS3* | Potri.005G078700 | Myo-inositol-1-phosphate synthase 3 | AT5G10170 | 5’-AACGTGCAATGCTTGAGAACAT-3’ | 5’-CAGAGAACCAGAGCAAACGG-3’ | 83 |
| *CYP76C6* | Potri.001G025200 | Cytochrome P450, family 76, subfamily C, polypeptide 6 | AT1G33720 | 5’-CCACTTCTTCTTCCTCACCGTG-3’ | 5’-GGGAACATGCTTGGTAGCCAT-3’ | 95 |
| *PS* | Potri.003G214500 | Peroxidase superfamily protein | AT5G19890 | 5'-GATCAAAATGGGGAATATAAGC-3' | 5'-CATGGGCTAAAACAAGACCT-3' | 80 |
| *SUS4* | Potri.006G136700 | Sucrose synthase 4 | AT3G43190 | 5'-GCTGCTGAACTCCTTGTTGACT-3' | 5'-CCGACTCAAACCAACCCA-3' | 84 |
| *LEA* | Potri.010G002600 | Late embryogenesis abundant domain-containing protein / LEA domain-containing protein | AT1G72100 | 5'-AAAGGCTGCTGAAACTCTGGAG-3' | 5'-GAGATGGGAAGCGAAGGAAGAT-3' | 85 |
| *GolS2* | Potri.013G005800 | Galactinol synthase 2 | AT1G56600 | 5'-GCCAATTCCTTCGGATTACAAC-3' | 5'-TGAAGTGGAGATCAACACCAGC-3' | 96 |
| *EXO* | Potri.002G098800 | Phosphate-responsive 1 family protein | AT4G08950 | 5’-TGCTTACATTTGGGTTGGTAAC-3' | 5'-GGCATAACCAGGATAGGCAC-3’ | 98 |
| *HS1* | Potri.010G150800 | Heat stable protein 1 | AT3G17210 | 5’-TAATCTGGGTATTCACGACCTC-3' | 5’-ACACCGCCAATTATTGCTTATA-3' | 100 |
| *WI12* | Potri.019G125900 | WOUND-INDUCED PROTEIN 12 | AT3G10985 | 5’-CATCTTCATCAACAAGACCAAG-3' | 5’-CTCTAGGCGGAGAAGTAATACC-3' | 97 |
| a |  | Actin 2/7 |  | 5’- CCCATTGAGCACGGTATTGT-3' | 5'-TACGACCACTGGCATACAGG -3’ | 89 |
| b |  | 18S rRNA |  | 5’-AGAAACGGCTACCACATCCAA-3' | 5’-CCAGACTTGCCCTCCAATGG-3' | 92 |
| a: From Brunner AM, Yakovlev IA, Strauss SH (2004) Validating internal controls for quantitative plant gene expression studies. BMC Plant Biol 4:14. | | | | | |  |
| b: From Junghans U, Polle A, Düchting P, Weiler E, Kuhlman B, Gruber F, Teichmann T (2006) Adaptation to high salinity in poplar involves changes in xylem anatomy and auxin physiology. Plant Cell Environ 29: 1519–1531 | | | | | |  |

**Table S10** The harvested leaf number, maximum root length (cm) and biomass of roots and leaves (g^-1^ DW) of *P. simonii* treated at either ambient (A) or high (H) temperatures combined with one of two watering regimes (well-watered (W) or drought (D)). Data indicate means ± SE (n = 6). Different letters after values in the same column indicate significant differences. *P*-values according to analysis of variance (ANOVA) tests for temperature (T), drought (D), and their interaction (T × D) are also indicated. *: *P* < 0.05; **: *P* < 0.01; ***: *P* < 0.001; ****: *P* < 0.0001; ns: not significant.

| Treatment | Leaf number | Maximum root length | Leaf biomass | Root biomass |
| --- | --- | --- | --- | --- |
| AW | 32.0 ± 0.6 a | 31.3 ± 0.8 a | 6.9 ± 0.9 b | 1.4 ± 0.1 a |
| AD | 31.7 ± 1.5 a | 32.5 ± 2.1 a | 5.5 ± 0.1 ab | 1.3 ± 0.5 a |
| HW | 29.0 ± 0.6 a | 30.8 ± 1.7 a | 6.6 ± 0.1 b | 1.7 ± 0.4 a |
| HD | 32.7 ± 1.7 a | 30.4 ± 2.6 a | 4.4 ± 0.8 a | 2.1 ± 0.1 a |
| T | ns | ns | ns | ns |
| D | ns | ns | * | ns |
| T × D | ns | ns | ns | ns |

**Figure S1** The fold changes of gene expression detected by RNA-seq (shown as dots) and by quantitative RT-PCR (shown as histograms). Quantitative RT-PCR results are expressed on the basis of Actin (a) and 18S rRNA (b) genes. Bars indicate means ± SE (n = 6).

**
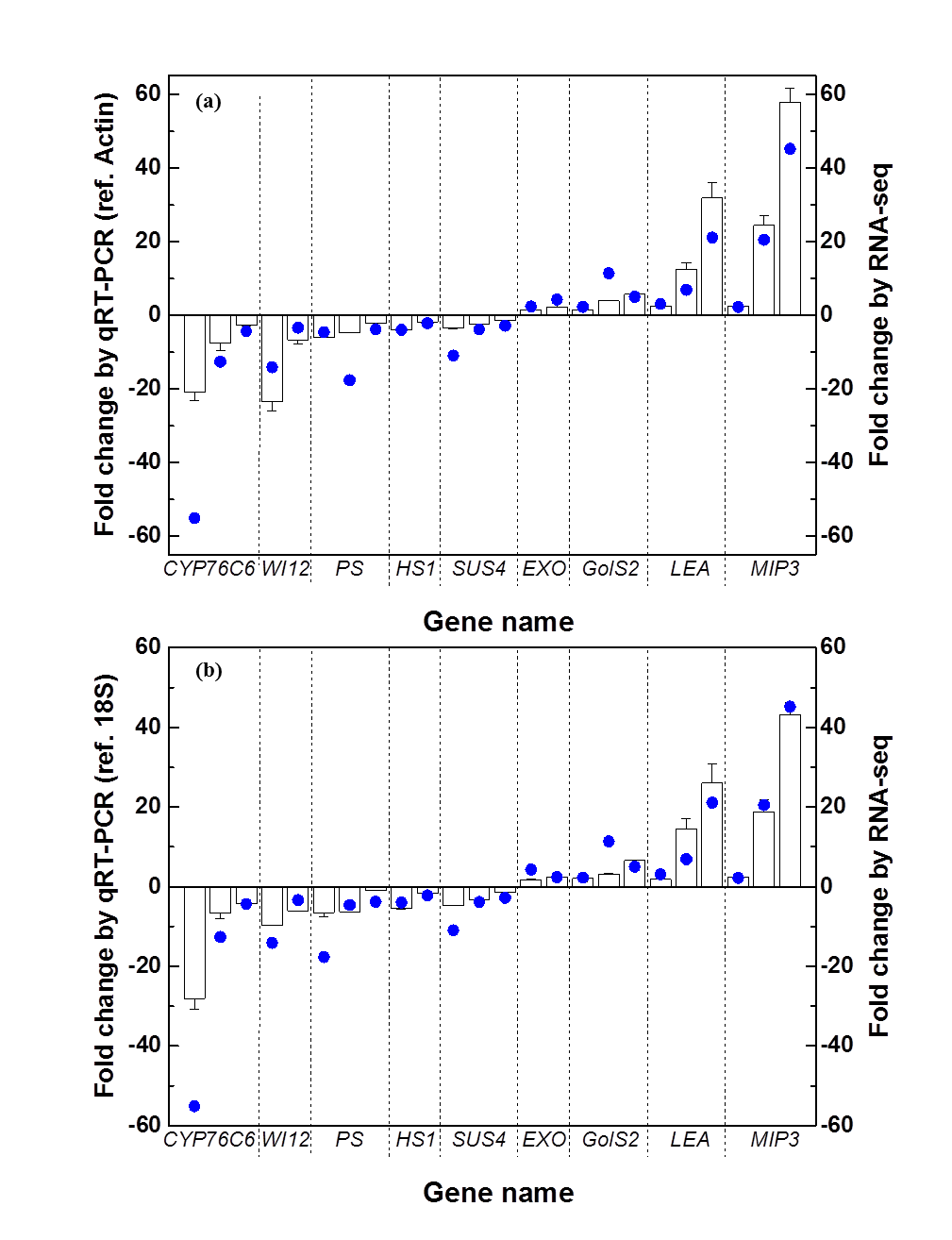
**

**Figure S2** The co-expression networks of drought responsive genes in the roots (a) and leaves (b) of *P. simonii*. An edge indicates the co-expression between the two genes. Red and blue nodes represent up- and down-regulated genes, respectively. The presence of each node in the network is indicated in Supplementary Table S5.

**
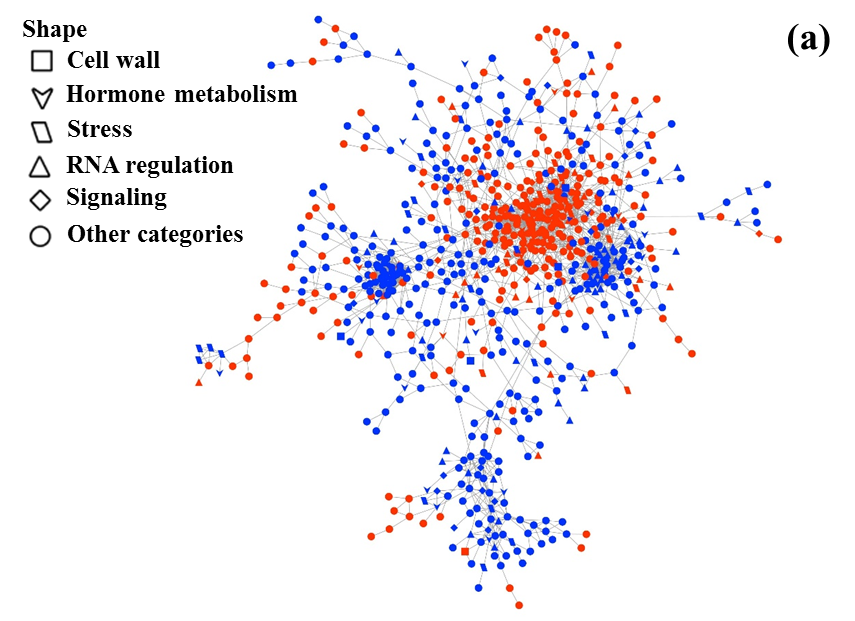
**

**
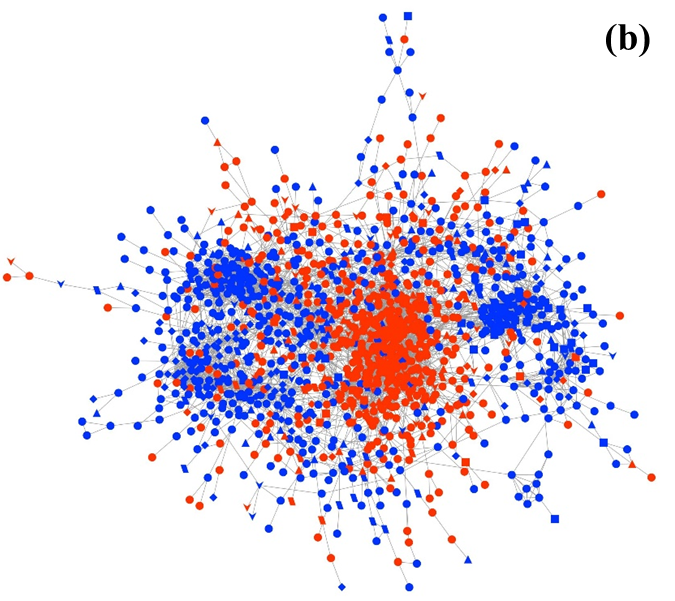
**

**Figure S3** Stomatal conductance (***g_s_***) during temperature and drought treatments in mature leaves of *P. simonii* exposed to either ambient (A) or high (H) temperatures combined with one of two watering regimes (well-watered (W) and drought (D)). Data indicate means ± SE (n = 3).

**Figure S4.** *δ^13^C*, *δ^15^N*, and *δ^18^O* in the roots (a–c) and leaves (d–f) of *P. simonii* treated at either ambient (A) or high (H) temperatures combined with one of two watering regimes (well-watered (W) or drought (D)). Bars indicate means ± SE (n = 6). Different letters on the bars indicate significant differences. *P*-values obtained from ANOVAs based on temperature (T), drought (D), and their interactions (T × D) are also indicated. *: *P* < 0.05; **: *P* < 0.01; ***: *P* < 0.001; ****: *P* < 0.0001; ns: not significant.


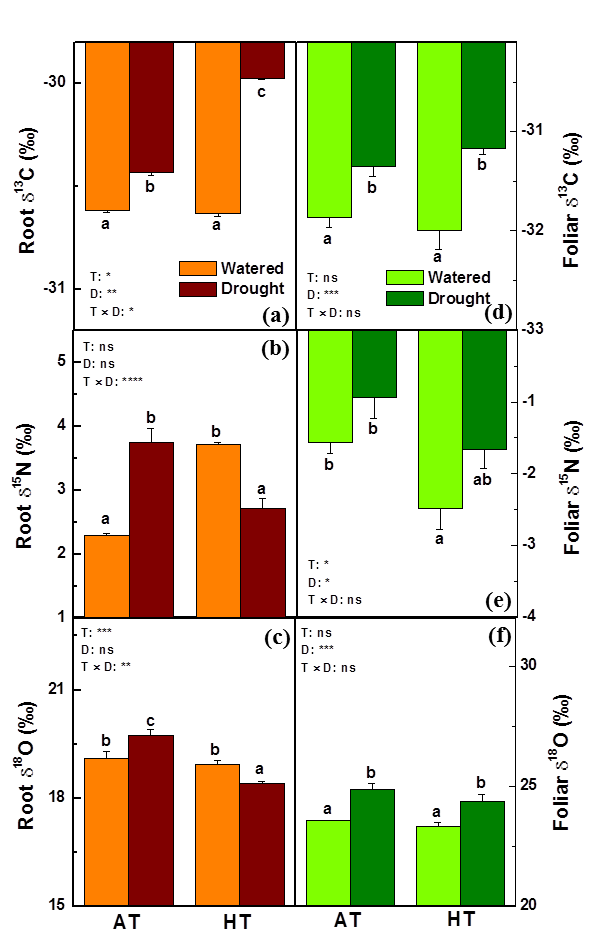


**Figure S5** The concentrations of ASC, DHA, GSH, and GSSG in the roots (a-d) and leaves (e-h) of *P. simonii* treated at either ambient (A) or high (H) temperatures combined with one of two watering regimes (well-watered (W) and drought (D)). Bars indicate means ± SE (n = 6). Different letters on the bars indicate significant differences. *P*-values according to analysis of variance (ANOVA) tests for temperature (T), drought (D), and their interaction (T × D) are also indicated. *: *P* < 0.05; **: *P* < 0.01; ***: *P* < 0.001; ****: *P* < 0.0001; ns: not significant.

**
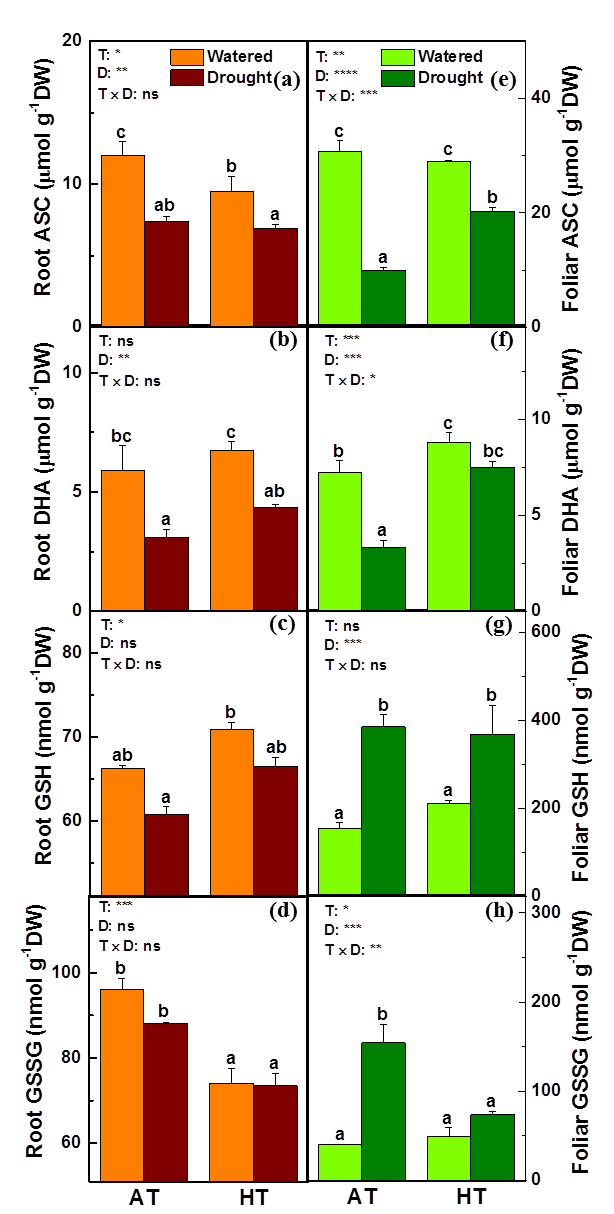
**

**Figure S6** Activities of superoxide dismutase (SOD), catalase (CAT), ascorbate peroxidase (APX), and glutathione reductase (GR) in the roots (a-d) and leaves (e-h) of *P. simonii* treated at either ambient (A) or high (H) temperatures combined with one of two watering regimes (well-watered (W) and drought (D)). Bars indicate means ± SE (n = 6). Different letters on the bars indicate significant differences. *P*-values according to analysis of variance (ANOVA) tests for temperature (T), drought (D), and their interaction (T × D) are also indicated. *: *P* < 0.05; **: *P* < 0.01; ***: *P* < 0.001; ****: *P* < 0.0001; ns: not significant.

**
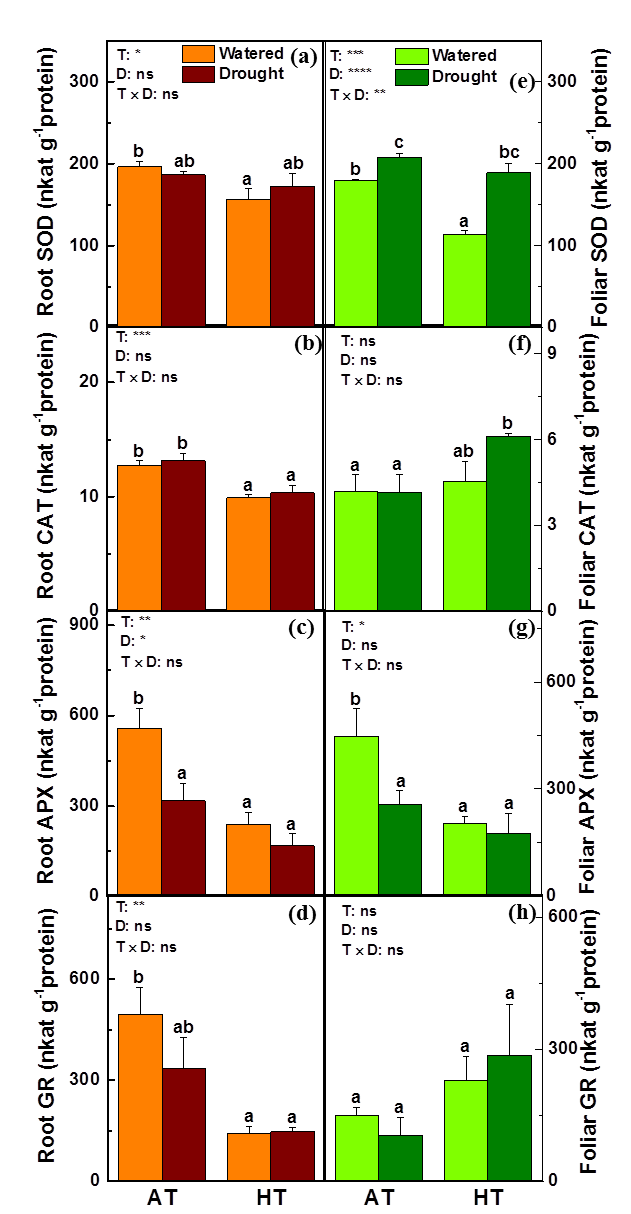
**

**Figure S7** Alignments of Myo-inositol-1-phosphate synthase 3 (MIPS3), Cytochrome P450 (CYP76C6), Peroxidase superfamily protein (PS), Sucrose synthase 4 (SUS4), Late embryogenesis abundant domain-containing protein / LEA domain-containing protein (LEA), Galactinol synthase 2 (GolS2), Phosphate-responsive 1 family protein (EXO), Heat stable protein 1 (HS1), and WOUND-INDUCED PROTEIN 12 (WI12) at the cDNA level and amino acid level among *Populus simonii* (Ps), *Populus trichocarpa* (Pt) and *Arabidopsis thaliana* (At).


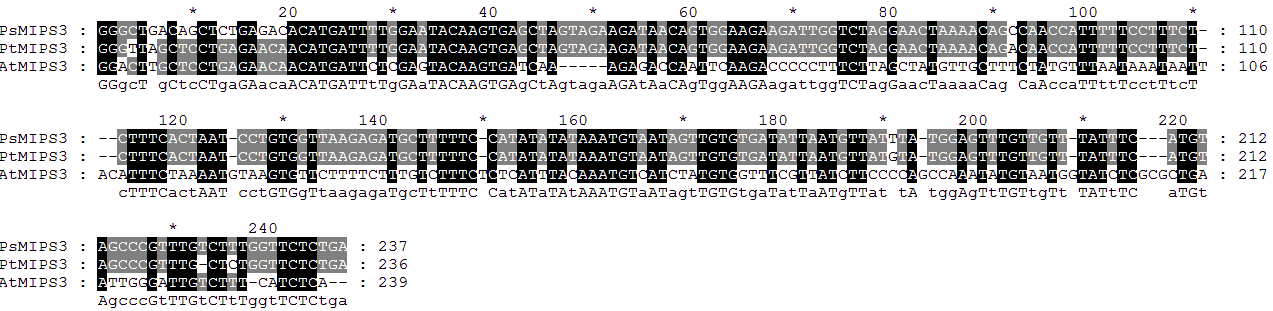


| Alignments at the cDNA level, *Populus trichocarpa* as a reference | | | | | | |
| --- | --- | --- | --- | --- | --- | --- |
|  |  |  |  |  | Gene ID |  |
| Identity for MIPS3 | Ps | 224/236*100 % = 94.9% |  | PtMIPS3 | Potri.005G078700 | |
|  | At | 121/236*100 % = 51.3% |  | AtMIPS3 | AT5G10170 | |

The primers of *MIPS3* were designed in 3'UTR. Thus, no amino acid sequence was available for alignment.


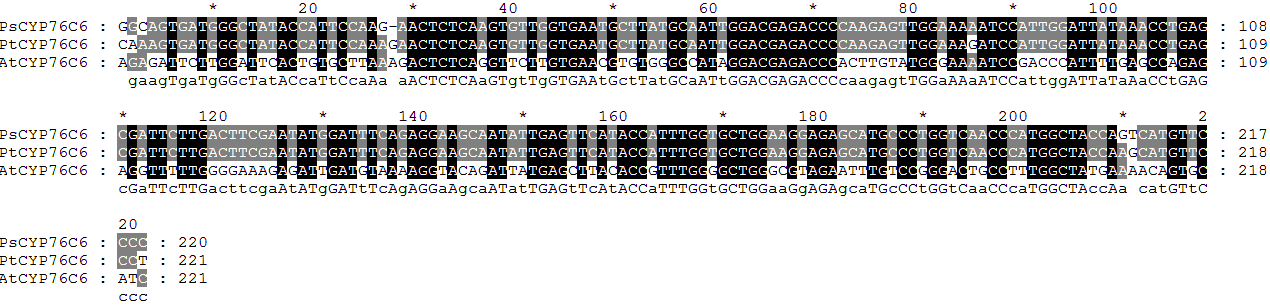


| Alignments at the cDNA level, *Populus trichocarpa* as a reference | | | | | |
| --- | --- | --- | --- | --- | --- |
|  |  |  |  |  | Gene ID |
| Identity for CYP76C6 | Ps | 212/221*100 % = 95.9% |  | PtCYP76C6 | Potri.001G025200 |
|  | At | 127/221*100 % = 57.5% |  | AtCYP76C6 | AT1G33720 |

The primers of *CYP76C6* were designed in 3'UTR. Thus, no amino acid sequence was available for alignment.


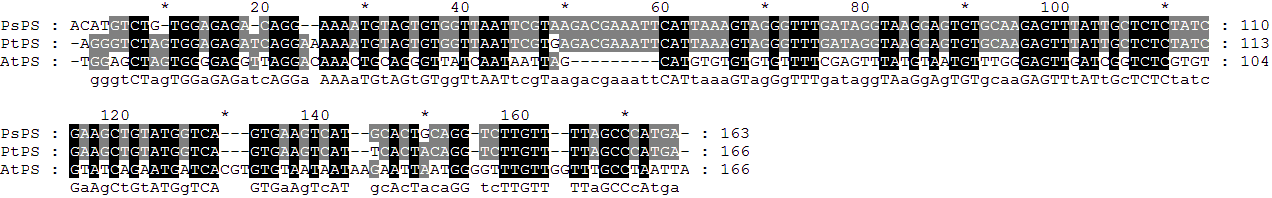


| Alignments at the cDNA level, *Populus trichocarpa* as a reference | | | | | |
| --- | --- | --- | --- | --- | --- |
|  |  |  |  |  | Gene ID |
| Identity for PS | Ps | 154/166*100 % = 92.8% |  | PtPS | Potri.003G214500 |
|  | At | 83/166*100 % = 50.0% |  | AtPS | AT5G19890 |

The primers of *PS* were designed in 3'UTR. Thus, no amino acid sequence was available for alignment.


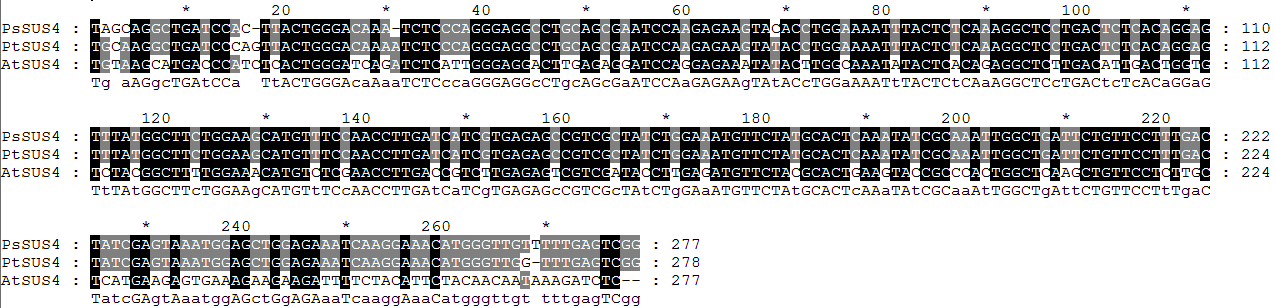


| Alignments at the cDNA level, *Populus trichocarpa* as a reference | | | | | |
| --- | --- | --- | --- | --- | --- |
|  |  |  |  |  | Gene ID |
| Identity for SUS4 | Ps | 268/278*100 % = 96.4% |  | PtSUS4 | Potri.006G136700 |
|  | At | 179/278*100 % = 64.3% |  | AtSUS4 | AT3G43190 |


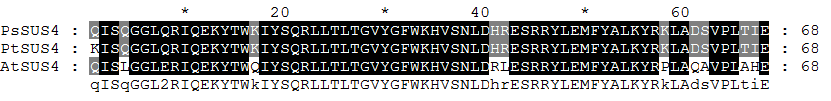


| Alignments at the amino acid level, *Populus trichocarpa* as a reference | | | | | |
| --- | --- | --- | --- | --- | --- |
|  |  |  |  |  | Gene ID |
| Identity for SUS4 | Ps | 67/68*100 % = 98.5% |  | PtSUS4 | Potri.006G136700 |
|  | At | 58/68*100 % = 85.3% |  | AtSUS4 | AT3G43190 |


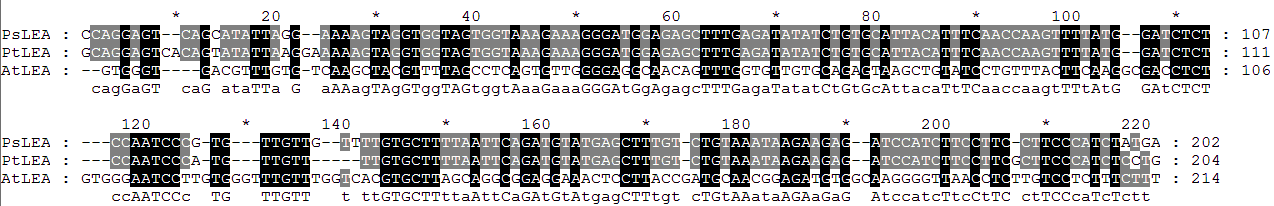


| Alignments at the cDNA level, *Populus trichocarpa* as a reference | | | | | |
| --- | --- | --- | --- | --- | --- |
|  |  |  |  |  | Gene ID |
| Identity for LEA | Ps | 188/204*100 % = 92.2% |  | PtLEA | Potri.010G002600 |
|  | At | 88/204*100 % = 43.1% |  | AtLEA | AT1G72100 |

The primers of *LEA* were designed in 3'UTR. Thus, no amino acid sequence was available for alignment.


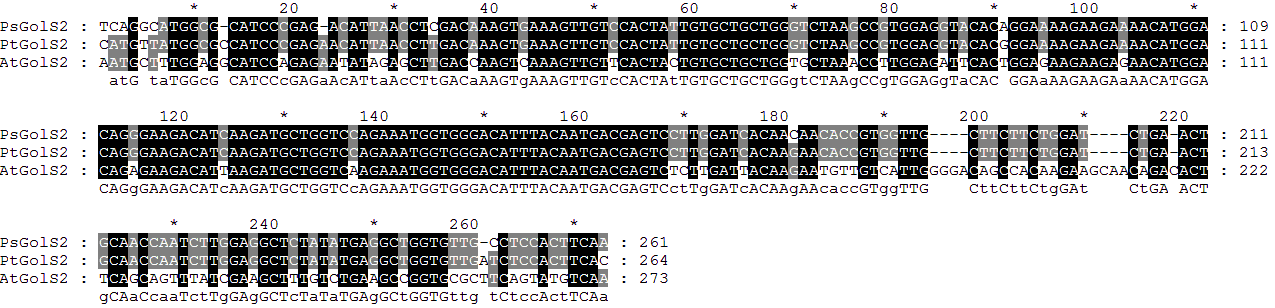


| Alignments at the cDNA level, *Populus trichocarpa* as a reference | | | | | |
| --- | --- | --- | --- | --- | --- |
|  |  |  |  |  | Gene ID |
| Identity for GolS2 | Ps | 251/264*100 % = 95.1% |  | PtGolS2 | Potri.013G005800 |
|  | At | 187/264*100 % = 70.8% |  | AtGolS2 | AT1G56600 |


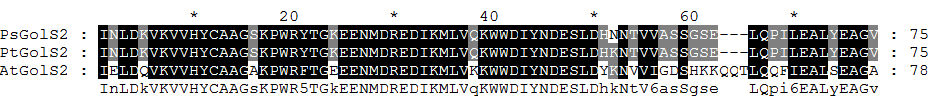


| Alignments at the amino acid level, *Populus trichocarpa* as a reference | | | | | |
| --- | --- | --- | --- | --- | --- |
|  |  |  |  |  | Gene ID |
| Identity for GolS2 | Ps | 74/75*100 % = 98.7% |  | PtGolS2 | Potri.013G005800 |
|  | At | 62/75*100 % = 82.7% |  | AtGolS2 | AT1G56600 |


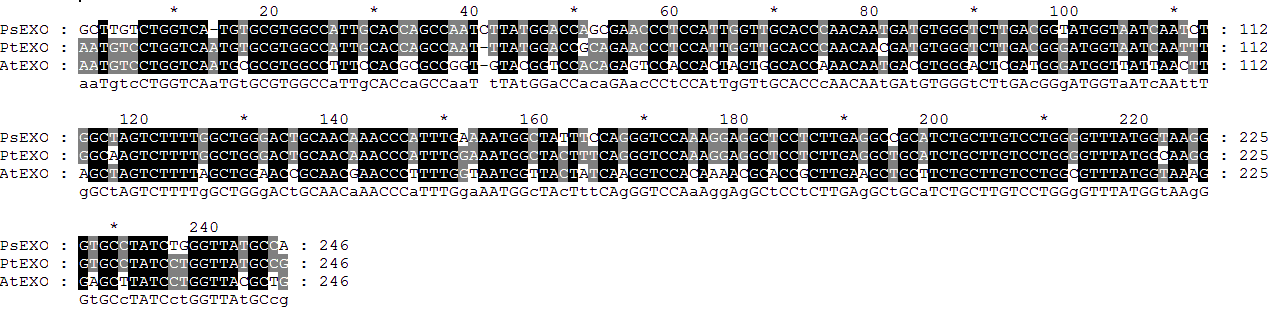


| Alignments at the cDNA level, *Populus trichocarpa* as a reference | | | | | |
| --- | --- | --- | --- | --- | --- |
|  |  |  |  |  | Gene ID |
| Identity for EXO | Ps | 224/246*100 % = 91.1% |  | PtEXO | Potri.002G098800 |
|  | At | 193/246*100 % = 78.5% |  | AtEXO | AT4G08950 |


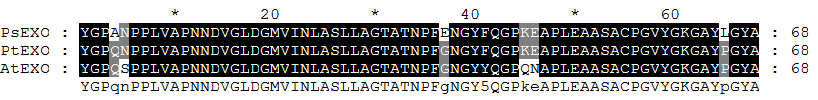


| Alignments at the amino acid level, *Populus trichocarpa* as a reference | | | | | |
| --- | --- | --- | --- | --- | --- |
|  |  |  |  |  | Gene ID |
| Identity for EXO | Ps | 65/68*100 % = 95.6% |  | PtEXO | Potri.002G098800 |
|  | At | 65/68*100 % = 95.6% |  | AtEXO | AT4G08950 |


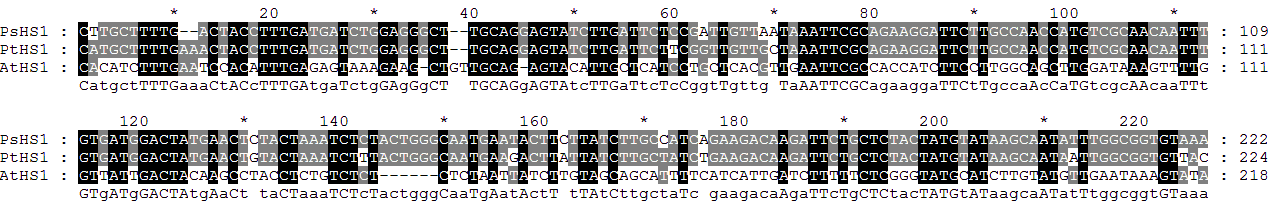


| Alignments at the cDNA level, *Populus trichocarpa* as a reference | | | | | |
| --- | --- | --- | --- | --- | --- |
|  |  |  |  |  | Gene ID |
| Identity for HS1 | Ps | 208/224*100 % = 92.9% |  | PtHS1 | Potri.010G150800 |
|  | At | 104/224*100 % = 46.4% |  | AtHS1 | AT3G17210 |

The primers of *HS1* were designed in 3'UTR. Thus, no amino acid sequence was available for alignment.


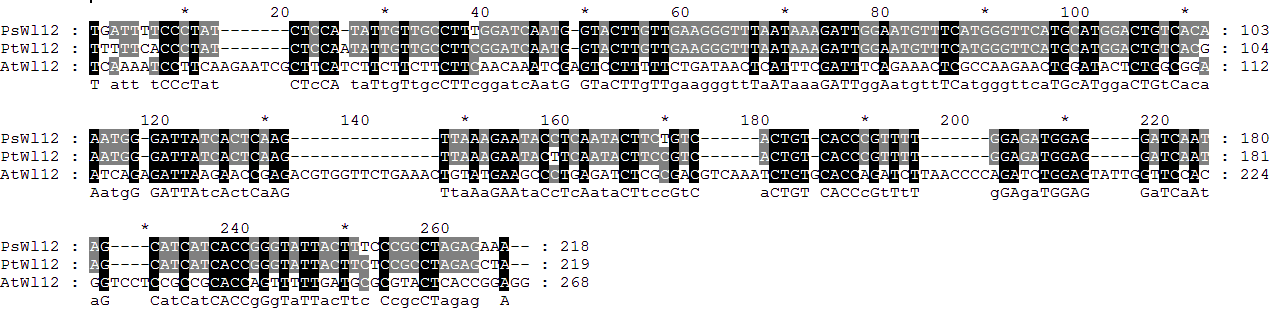


| Alignments at the cDNA level, *Populus trichocarpa* as a reference | | | | | |
| --- | --- | --- | --- | --- | --- |
|  |  |  |  |  | Gene ID |
| Identity for Wl12 | Ps | 206/219*100 % = 94.1% |  | PtWl12 | Potri.019G125900 |
|  | At | 84/219*100 % = 38.4% |  | AtWl12 | AT3G10985 |


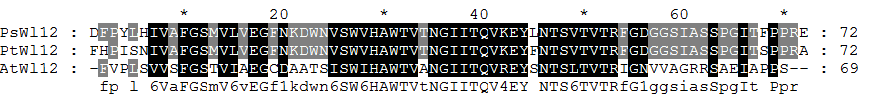


| Alignments at the amino acid level, *Populus trichocarpa* as a reference | | | | | |
| --- | --- | --- | --- | --- | --- |
|  |  |  |  |  | Gene ID |
| Identity for Wl12 | Ps | 66/72*100 % = 91.7% |  | PtWl12 | Potri.019G125900 |
|  | At | 42/72*100 % = 58.3% |  | AtWl12 | AT3G10985 |
